# Lung Immune Cell Niches and the Discovery of New Cell Subtypes

**DOI:** 10.1002/advs.202405490

**Published:** 2024-10-14

**Authors:** Qing'e Shan, Jiahuang Qiu, Zheng Dong, Xiaotong Xu, Shuping Zhang, Juan Ma, Sijin Liu

**Affiliations:** ^1^ Medical Science and Technology Innovation Center Shandong First Medical University & Shandong Academy of Medical Sciences Jinan Shandong 250117 P. R. China; ^2^ State Key Laboratory of Environmental Chemistry and Ecotoxicology Research Center for Eco‐Environmental Sciences Chinese Academy of Sciences Beijing 100085 P. R. China; ^3^ School of Public Health Shandong First Medical University & Shandong Academy of Medical Sciences Jinan Shandong 250117 P. R. China; ^4^ Dongguan Key Laboratory of Environmental Medicine School of Public Health Guangdong Medical University Dongguan 523808 P. R. China; ^5^ School of Environmental Sciences University of Chinese Academy of Sciences Beijing 100049 P. R. China

**Keywords:** cell‐cell crosstalk, lung development, lung immune cells, new immune cell subtypes

## Abstract

Immune cells in the lungs are important for maintaining lung function. The importance of immune cells in defending against lung diseases and infections is increasingly recognized. However, a primary knowledge gaps in current studies of lung immune cells is the understanding of their subtypes and functional heterogeneity. Increasing evidence supports the existence of novel immune cell subtypes that engage in the complex crosstalk between lung‐resident immune cells, recruited immune cells, and epithelial cells. Therefore, further studies on how immune cells respond to perturbations in the pulmonary microenvironment are warranted. This review explores the processes behind the formation of the immune cell niche during lung development, and the characteristics and cell interaction modes of several major lung‐resident immune cells. It indicates that distinct lung microenvironments or inflammatory niches can mediate the formation of different cell subtypes. These findings summarize and clarify paths to identify new cell subtypes that originate from resident progenitor cells and recruited peripheral cells, which are remodeled by the pulmonary microenvironment. The development of new techniques combining transcriptome analysis and location information is essential for identifying new immune cell subtypes and their relative immune niches, as well as for uncovering the molecular mechanisms of immune cell‐mediated lung homeostasis.

## Graphical abstract

This review clarified that progenitor cells from early lung development stages and peripheral cells recruited into the lungs can be remodeled by the pulmonary microenvironment, leading to the emergence of new cell subtypes and the establishment of specific immune niches. Furthermore, advancements in single‐cell RNA sequencing and spatial transcriptomics are crucial for uncovering these new immune cell subsets. The figure was created with BioRender.com (https://biorender.com).

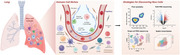

## 1 Introduction

The lungs are the initial target of damage for inhaled substances such as dust, smoke, pollen, and aerosols, as well as respiratory bacteria and viruses.^[^ ^1^ ^]^ Immune cells play a crucial role in clearing of pathogenic factors and managing intense inflammation, but they can also elevate the risk of functional disorders and tissue damage. To preserve proper lung function, immune cells within the lungs must maintain a delicate balance between mounting an immune response and exercising tolerance. The homeostasis is mainly mediated by the complex regulatory network involving non‐circulating resident immune cell subsets and the initial cells recruited from circulation, particularly tissue‐resident cells that provide rapid and initial immune protection through both innate and adaptive immunity. For instance, alveolar macrophages (AMs) are the most studied cells for maintain lung homeostasis and for initiating the recruitment of other immune cells.^[^ ^2^ ^]^ However, the other types of resident immune cells in the lungs and the mechanisms behind their maintenance functions mechanism are not fully understood. Furthermore, the types of immune cells recruited into the lungs and the specific cell subtypes, whose functional transformations are mediated by the pulmonary immune microenvironment, remain unclear.

The development of the immune system occurs in the early embryo, and the heterogeneity of immune cells in the lungs increases rapidly at birth. Therefore, understanding how dynamic physiological changes in lung immune cells before and after birth alter the immune cell landscape in the lungs is important. Although innate immune cells residing in the lungs are considered the first line of defense against infections, their origins, and self‐renewal mechanisms are not well understood. For instance, the late embryonic lung is dominated by specialized proliferative macrophages that interact physically with the developing vasculature. After birth, these macrophages disappear and are replaced by a dynamic mixture of macrophage subtypes, dendritic cells (DCs), granulocytes, and lymphocytes.^[^ ^3^ ^]^ It remains unclear whether these new immune cells originate from macrophages or other extrapulmonary sources.^[^ ^3^ ^]^ Evidence suggests that the composition of the immune microenvironment is highly plastic, owing to dynamic changes in immune cell sub‐populations. When extrapulmonary immune cells are recruited to the lungs, they gradually adapt to the pulmonary microenvironment and differentiate into cell subsets with different functions. Therefore, the molecular regulatory mechanism that underlie the activation of innate immune cells and their induction of adaptive immune responses warrant further study. Such knowledges will also provide additional clues for potentially discovering new immune cell subsets that contribute to the regulation of specific immune responses in infectious and non‐infectious diseases.^[^ ^4^ ^]^

In this review, we propose that lung‐resident immune cells, derived from progenitor cells, contribute to the formation of the primary immune niche during lung organogenesis. The lung's unique anatomy facilitates the maturation of immune cells and serves as an efficient site for direct cell‐cell communication, which is essential for the emergence of distinct and novel immune subtypes. We highlight that immune cells recruited from the circulating blood undergo significant remodeling within the intrapulmonary microenvironment. This process results in the formation of a new immune niche through interactions among specific immune cells, cytokines, chemokines, and lung tissue cells. The niche, defined by its specific location, mediates the function of other recruited cells and promotes the emergence of new immune cell subtypes. Notably, the advent of sophisticated single‐cell RNA sequencing (scRNA‐seq) techniques, particularly when integrated with spatial information, has laid a robust technical groundwork for the identification of novel immune subsets within the lungs.

## 2 Stages of Lung Development and Dominant Composition of the Cellular Niche

**Figure 1:**
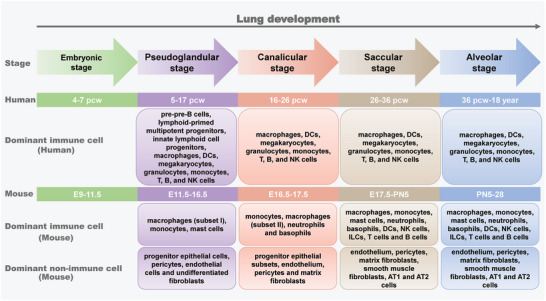
Stages of lung development and dominant composition of lung cells. pcw: post‐conception weeks; E: embryonic; PN: postnatal; DCs: dendritic cells; NK cells: natural killer cells; ILCs: innate lymphoid cells.

The development process of the lung has been identified as being composed of five morphologically distinct stages (**Figure** **1**). It is important to note that the developmental stages of human and mouse lungs are categorized in chronological order as embryonic, pseudoglandular, canalicular, saccular, and alveolar stages, with differences in the timing of these stages.^[^ ^5^ ^]^ Figure 1 provides a detailed summary of the specific temporal divisions and cell subtypes present in each stage.

The embryonic stage marks the initial stage of lung development, characterized by the appearance of lung buds. Subsequently, the pseudoglandular stage primarily involves the development of the main respiratory tract and the formation of terminal bronchi.^[^ ^6^ ^]^ The canalicular stage is characterized by the appearance of acini, formation of the air‐blood barrier, differentiation of alveolar type 1 cells (AT1s) and alveolar type 2 cells (AT2s), and the gradual secretion of surfactant.^[^ ^7^ ^]^ The saccular phase is characterized by the formation of alveolar vesicle clusters in the alveolar duct and accelerated surfactant production.^[^ ^8^ ^]^ The alveolar stage primarily involves alveolar development and maturation. With the development and maturation of lung tissue structures, lung function tends to gradually mature as well.^[^ ^9^ ^]^

In mice, the development of CD45^+^ immune cell component begins during the early stage of morphogenesis (E12.5). At this stage, the immune cell type is predominantly made up of macrophages (subset Ι, which constitute over 50% of CD45^+^ cells), monocytes (10%) and mast cells (11%). In the canalicular stage, the predominant cell types are monocytes, macrophages (subset ΙΙ), neutrophils, and basophils，with monocytes being the most abundant, making up 58% of the total. Moving on the saccular stage, all major immune cell groups are found in the lungs including macrophages, monocytes, mast cells, neutrophils, basophils, DCs, natural killer (NK) cells, innate lymphoid cells (ILCs), T cells and B cells. Subsequently, the lymphoid cell compartment (comprised of B and T cells) increased steadily and finally reaching 32% of the immune cell population on the 7th day following birth.^[^ ^10^ ^]^

Regarding the development of non‐immune cells (CD45^−^) in mice, the early stage of morphogenesis (E12.5) is primarily populated by undifferentiated fibroblasts, constituting 83% of CD45^−^ cells, and progenitor epithelial cells, which make up 10%. Additionally, a small number of pericytes and endothelial cells are present. As development progresses to the canalicular stage, the proportion of progenitor epithelial subsets increases, reaching up to 30%, alongside an increase in the endothelium and the emergence of pericytes and matrix fibroblasts. Notably, smooth muscle fibroblasts appear, and epithelial cells differentiate into AT1 and AT2 cells during the saccular stage. Moreover, from the saccular stage onwards, the components of non‐immune cell types begin to stabilize.^[^ ^6^, ^10^ ^]^

Barnes et al. discovered that immune cells in the lungs of human fetuses evolve with developmental progress.^[^ ^11^ ^]^ Certain early progenitor cells are predominantly found during the pseudoglandular stage, including pre‐pre‐B cells, lymphoid‐primed multipotent progenitors, innate lymphoid cell progenitors, megakaryocyte‐erythroid progenitors, common myeloid progenitors, megakaryocyte progenitors, and T cell progenitors. Additionally, macrophages, mast cells, and NK cells are also present during this stage.^[^ ^11^ ^]^ It is also noteworthy that myeloid cells—such as macrophages, DCs, megakaryocytes, and granulocytes—begin to appear from 8 post‐conception weeks (pcw), while monocytes gradually increase from 15 pcw, and T, B, and NK cells become prominent from 15 pcw.^[^ ^11^, ^12^ ^]^ Overall, the proportion of immune cells follows a biphasic pattern, peaking at 8 pcw and rising again at 20 pcw.^[^ ^11^ ^]^ Research conducted a thorough examination of cellular heterogeneity within human embryonic lung, indicating that the diversity of gene expression patterns in the developing human lung may stem from the distinct gene expression programs of progenitor cells from different germ layers for various cell categories, the regional‐specific gene expression profiles of some cell clusters, and the cellular communication patterns within local environments.^[^ ^13^ ^]^

Overall, the formation of specialized cell types during lung development relies on highly regulated processes and microenvironments. The lung provides a unique anatomical site for immune cell to mature and function as efficient site for direct cell‐cell communications, which are required for the induction of unique immune subtypes. Understanding the composition of cell types in the lungs, particularly the discovery of new cell subsets, can potentially reveal the cell‐cell crosstalk that occurs during pathophysiological processes.

## 3 Resident Immune Cells in the Lung and their Potential New Subtypes

### 3.1 Macrophages

Studies have shown that macrophage development in the lung goes through at least two stages.^[^ ^14^ ^]^ In the first stage, primitive macrophages develop from the fetal yolk sac, independent of the formation of monocyte intermediates. Additionally, most tissue‐resident macrophage populations originate from fetal monocyte precursors.^[^ ^14b^ ^]^ With the first breath following birth, tissue‐resident macrophages in the lungs begin to differentiate into resident AMs and interstitial macrophages (IMs) based on their original locations.^[^ ^1^, ^14^, ^15^ ^]^ In the stable state, most AMs reside in the alveoli, while IMs reside in the lung parenchyma.^[^ ^16^ ^]^ There are two main sources of macrophage origin: one is derived from embryonic or fetal progenitor cells that are mainly locally self‐renewed, and the other is derived from adult monocytes that develop from hematopoietic stem and progenitor cells.^[^ ^17^ ^]^

#### 3.1.1 AMs

AMs primarily colonize the alveolar lumen and constitute 90–95% of the cells within the alveoli under steady‐state conditions. Despite mice possessing millions of alveoli, their lungs house only ≈1–2 million AMs. Although one AM can be found per three alveoli, these macrophages are capable of traversing between adjacent alveoli via the pores of Kohn.^[^ ^18^ ^]^

L‐plastin, a protein that regulates the actin cytoskeleton, plays an indispensable role in the migration of mouse macrophage precursors to the alveolar lumen.^[^ ^19^ ^]^ In addition, granulocyte‐macrophage colony‐stimulating factor (GM‐CSF), transforming growth factor‐β (TGF‐β), and peroxisome proliferator‐activated receptor γ (PPARγ) mediate the differentiation of embryonic monocytes into mature AMs after birth.^[^ ^15^, ^20^ ^]^ The contribution of monocytes derived from hematopoietic progenitor cells to the murine AM pool increases steadily with age. Mice deficient in AMs due to a lack of GM‐CSF, specifically *Csf2* ^−/−^ mice, or its receptor GM‐CSFR, specifically *Csf2rb* ^−/−^ mice, exhibit impaired viral clearance and increased mortality following influenza virus infection.^[^ ^21^ ^]^ Additionally, the overexpression of GM‐CSF in lung tissue can prevent AM apoptosis and enhance resistance to influenza.^[^ ^22^ ^]^

**Figure 2:**
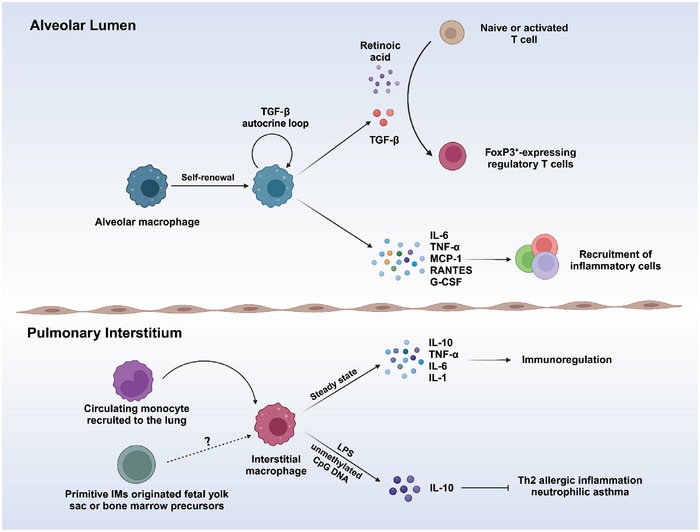
Self‐renewal and activation mechanisms of AMs and IMs. AMs and IMs are resident macrophages in the lungs that can not only protect the lungs from inhaled pathogens and allergens, but also prevent excessive immune response to harmless particles. The maintenance of AMs depends on the self‐renewal of resident macrophages in the alveolar lumen. AMs exert their anti‐inflammatory effects by secreting TGF‐ β, which restricts the activation of AM through the autocrine loop. AMs also provide protection against inhaled pathogens by secreting a number of cytokines and chemokines—including interleukin‐6 (IL‐6), tumor necrosis factor‐α (TNF‐α), monocyte chemoattractant protein 1, RANTES, and granulocyte colony‐stimulating factor (G‐CSF). IMs located in the lung interstitium are mainly derived from recruited circulating monocytes. They play a key role in pulmonary immunoregulation and represent an important source of steady‐state immunoregulatory cytokines such as IL‐10, TNF‐α, IL‐6, and IL‐1. In addition, IL‐10 produced by IMs may be involved in Th2 allergic inflammation and neutrophil asthma.^[^ ^28^ ^]^ The figure was created with BioRender.com (https://biorender.com).

AMs are self‐renewing populations (**Figure** **2**) that do not depend on bone marrow (BM) replenishment.^[^ ^23^ ^]^ The self‐renewal of AMs occurs mainly through local proliferation and is largely independent of circulating monocytes during homeostasis.^[^ ^15^, ^24^ ^]^ Following non‐genotoxic depletion or infection disturbance of lung tissue macrophages, AMs can be locally replenished through self‐renewal, and their ability to produce cytokine colony‐stimulating factors 1 (Csf‐1) and 2 (Csf‐2) is enhanced.^[^ ^24b^ ^]^ New AMs derived from recruiting monocytes display a gradual transition wherein they become similar to tissue‐resident AMs, indicating that the recruited cells exhibit plasticity.^[^ ^24b^ ^]^

AM activation and the initiation of inflammatory responses both involve complex regulation between activation and inhibition signals. On one hand, surface toll‐like receptors (TLRs) combined with their co‐receptors (such as MD2 and CD14) trigger downstream signals such as tumor necrosis factor (TNF), interleukin‐1β (IL‐1β), and interferon‐γ (IFN‐γ), which recognize pathogen‐associated molecular patterns (PAMPs) and activate AMs to become more phagocytic.^[^ ^2^ ^]^ In return, these release large amounts of pro‐inflammatory factors to recruit other immune cells and promote inflammatory responses. On the other hand, the interactions between AMs and airway epithelial cells transmit inhibitory signals to AMs such as via CD200R, TREM2, or SIRP‐α. AMs initiate effective immune responses to protect against tissue injury during infection. By contrast, during inflammation resolution, the up regulation of negative regulatory factors and inhibition of TLR signaling pathways inhibit AM activation.^[^ ^2^ ^]^ Consequently, benefiting from their location in the alveolar lumen, AMs attach themselves to the pulmonary epithelium and sample microbes flowing with the alveolar fluid to serve as the first line of defense against various pathogens.^[^ ^25^ ^]^ The phagocytic capacity of activated AMs is often enhanced during states of infection,^[^ ^18a^ ^]^ such as those by *Streptococcus pneumoniae*, *Mycobacterium tuberculosis*, *Pseudomonas aeruginosa*, and others.^[^ ^26^ ^]^ AMs can engulf apoptotic epithelial cells and surfactant phospholipids that accumulate in alveoli during influenza virus infection and obstruct ventilation to restore the normal state after infection‐mediated injuries.^[^ ^27^ ^]^ Furthermore, AMs bolster the lung's defense against inhaled pathogens through the secretion of an array of cytokines and chemokines. This includes interleukin‐6 (IL‐6), TNF‐α, monocyte chemoattractant protein 1, regulated on activation, normal T cell expressed and secreted (RANTES), and granulocyte colony‐stimulating factor (G‐CSF).^[^ ^28^ ^]^

#### 3.1.2 IMs

IMs tend to be located in the pulmonary interstitium between the alveolar epithelium and capillaries, or in the alveolar interstitium, submucosal, or perivascular adventitia.^[^ ^29^ ^]^ Thus, the origin and ontogeny of IMs are complex phenomena that have not been definitively elucidated.^[^ ^14^, ^30^ ^]^ Compared with lung AMs, IMs have been poorly studied possibly because isolating them relies on digestion of lung tissue digestion. This leads to greater difficulty in obtaining single‐cell suspensions of the lung interstitium than bronchoalveolar lavage fluid (BALF). Lineage‐tracing experiments have indicated that primitive IMs initially originate from the fetal yolk sac during embryonic development, followed by a second wave of defined IMs that originate from bone marrow precursors. And a small proportion of these IMs (Figure 2) may persist into adulthood.^[^ ^14a^ ^]^ Adoptive transplantation experiments have shown that monocytes continue to migrate into lung tissues regardless of the inflammatory state,^[^ ^31^ ^]^ supporting the notion that IMs rely on monocyte replenishment in a steady state, unlike the self‐renewal mechanism of AMs.

Functionally, IMs may participate in pulmonary barrier immunity alongside AMs. IMs appear to possess a more effective antigen presentation ability than AMs, which enables them to facilitate both T cell proliferation and the differentiation of regulatory T cells (Tregs).^[^ ^32^ ^]^ Upon exposure to lipopolysaccharide, IMs become activated, exhibiting enhanced phagocytosis, chemotaxis and the generation of reactive oxygen species.^[^ ^33^ ^]^ IMs are pivotal in pulmonary immunoregulation and serve as a significant source of cytokines that maintain immune balance under steady‐state conditions, such as IL‐10, TNF‐α, IL‐6, and IL‐1. Notably, IL‐10, which is produced by IMs, may also participate in the regulation of Th2‐mediated allergic inflammation and neutrophilic asthma.^[^ ^28^ ^]^ Recent studies have shown that there are multiple IM subgroups in the lungs with different functions, phenotypes, and longevities.^[^ ^34^ ^]^ For example, Ginhoux et al. demonstrated that there are two monocyte‐derived IM populations—Lyve1^lo^ MHC II ^hi^ IMs and Lyve1^hi^ MHC II ^lo^ IMs—with different gene expression profiles, phenotypes, functions, and localizations in murine lungs.^[^ ^32^ ^]^ The different IM populations are mainly distributed in specific and relatively fixed tissue niches, with Lyve1^lo^ MHC II ^hi^ IM located near nerve fibers and Lyve1^hi^ MHC II ^lo^ IM located near blood vessels. The absence of Lyve1^hi^MHCII^lo^ IMs exacerbates pulmonary fibrosis, demonstrating their critical role in tissue inflammation.^[^ ^32^ ^]^ Regardless of their origin, IMs are an important source of stable immunomodulatory cytokines and play a crucial role in pulmonary immunomodulation.

### 3.2 Dendritic Cells

DC development is a continuous process that begins in the BM and continues as the cells mature in various peripheral tissues. Therefore, DCs are not strictly self‐replenishing, tissue‐resident population.^[^ ^16^, ^35^ ^]^ Considering the longevity and proliferative potential of DC precursors, it is necessary to classify lung DCs into distinct subgroups, even though they may not exhibit the same extremely sedentary lifestyle as AMs.^[^ ^35a^ ^]^ Lung DCs can be broadly divided into conventional DCs (cDCs) and plasmacytoid DCs (pDCs), where each subset represents an independent developmental lineage with distinct but overlapping functions.^[^ ^36^ ^]^

For cDC development, multipotent hematopoietic stem cells differentiate into DC‐restricted common DC precursor cells (CDPs) during early progenitor cell stages.^[^ ^37^ ^]^ CDPs further generate pre‐DCs that migrates from the BM to non‐hematopoietic tissues and locally differentiate into cDC subtypes—including peripheral secondary lymphoid tissue‐resident DCs and migratory or tissue‐resident CD103^+^ DCs.^[^ ^21^, ^38^ ^]^ The cytokine Flt3L (the ligand for the receptor tyrosine kinase Flt3) has been identified as a marker of different stages of DC development, inducing the differentiation and proliferation of pre‐DCs and mature cDCs in peripheral tissues.^[^ ^38^, ^39^ ^]^ The development of lung cDCs is similar to that of other peripheral organs, in that pre‐DCs migrate into lungs and locally differentiate into mature DCs.^[^ ^16^ ^]^ The development of cDC and pDC in mature lungs is also dependent on the cytokine Flt3L.^[^ ^38^, ^40^ ^]^ Previous studies have found the presence of cells similar to the pre‐DC phenotype in the lung, but there is no definitive evidence supporting the notion that these cells can form pulmonary cDCs in situ.^[^ ^28^ ^]^

The development of lung‐resident pDCs has not been elucidated in detail. Some preliminary mechanisms of pDC development nave demonstrated that pDC subsets are fully differentiated in the BM, before they migrate to peripheral lymphoid organs, which is quite different from the developmental pathway of cDCs.^[^ ^41^ ^]^ In the BM, cytokines such as macrophage colony‐stimulating factor, IL‐7, and thrombopoietin act synergistically with Flt3L to jointly regulate pDC development.^[^ ^42^ ^]^ In addition, other transcription factors also mediate the formation and maturation of pDCs—including IRF8 and STAT3 in the BM, and Runx2 in peripheral tissues.^[^ ^42^, ^43^ ^]^

DCs capture antigens in the lungs, migrates to draining lymph nodes, and are located in T cell‐enriched regions, where they deliver antigenic peptides to antigen‐specific T cells to initiate adaptive immune responses.^[^ ^44^ ^]^ In the lungs, DCs are primarily located on the basolateral side of the epithelium, where they sample antigens in the alveolar lumen and conducting airways.^[^ ^45^ ^]^ The sampling activity of DCs largely depends on their location in the lungs. DCs located in the alveolar lumen have many dendrites that contribute to the continuous capture of antigens, whereas DCs in conducting airways appear to sample antigens infrequently.^[^ ^46^ ^]^ In addition, there is an increasingly clear division of functions among lung DC subpopulations, based on their specific performance and overlapping functions under conditions of homeostasis and inflammation.^[^ ^47^ ^]^ For example, when inhaled allergens are present, the CD103 DC population is the main DC subgroup in the lung that triggers the Th2 response, while the CD11b^+ hi^ DC subgroup induces the Th1 response.^[^ ^47b^ ^]^

### 3.3 Natural Killer Cells

Lung NK cells play an indispensable role in the pathological process of respiratory diseases—including infectious diseases, allergies, and cancer. Increasing evidence has suggested that lung‐resident NK cells are functionally and phenotypically distinct from other NK cells.^[^ ^48^ ^]^

Studies have shown that human lung‐resident NK cells exhibit a highly differentiated phenotype characterized as CD56^dim^ CD16^+^ and display a low response to stimulation.^[^ ^49^ ^]^ However, the majority of these NK cells lack the tissue‐resident marker CD69, suggesting that they mainly recirculate rather than residing permanently in the lungs.^[^ ^50^ ^]^ In mice, NK cells are a recirculating population, in contrast to tissue‐resident ILCs in the lungs. However, other studies have found evidence of functional and potentially resident CD49a^+^CD103^+^CD69^+^ NK subgroups in human lung explants, which are present in the parenchyma and degranulate upon viral infection.^[^ ^51^ ^]^ However, researchers have found that there are resident NK subsets with the CD49a^+^CD103^+^CD69^+^ phenotype in the parenchyma of human lungs that degranulate during viral infection.

Under steady‐state conditions, NK cells account for ∼10% of lymphocytes in the lungs. These lung NK cells are mainly CD27^lo^ subpopulations, which have highly mature phenotypes and self‐recognition inhibitory receptors.^[^ ^52^ ^]^ In addition to the widely recognized role in controlling tumor growth and metastasis, studies have confirmed the importance of lung‐resident NK cells in curbing the progression of lung neoplasms. These findings suggest that, compared to migratory NK cells, lung‐resident NK cells play a more dominant role in regulating tumor growth and metastasis. Similar to ILC1s, lung NK cells may contribute to pathogenesis of chronic lung inflammatory diseases, such as chronic obstructive pulmonary disease (COPD) and asthma, through the production of proinflammatory cytokines.

### 3.4 Innate Lymphoid Cells

ILCs initially develop in the fetal liver and later enter the adult BM for further development. Common lymphoid progenitor cells, derived NK cell precursors, and common helper innate lymphoid precursors subsequently differentiate into three subsets—dubbed group 1 ILCs (ILC1s), group 2 ILCs (ILC2s) and group 3 ILCs (ILC3s)—under the regulation of different transcription factors and interleukins.^[^ ^53^ ^]^ ILCs have an extremely sedentary lifestyle, and can be maintained via self‐renewal in various tissues, enabling them to sever as sentinels and local guardians to maintain tissue function.^[^ ^50^, ^53^, ^54^ ^]^ Notably, tissue‐resident ILCs use plasticity to reshape their phenotypes and functions in response to regulatory factors from their surrounding microenvironments.^[^ ^55^ ^]^ IL‐12 and IL‐15 can activate ILC1s and promote IFN‐γ production in this population. IL‐25, IL‐33 and thymic stromal lymphopoietin (TSLP) activate ILC2s and induce ILC2s to secrete type 2 cytokines such as IL‐5 and IL‐13 to mediate tumor killing. IL‐23 and IL‐1β are essential for the activation of ILC3s, but vitamin A deficiency may affect the amount and function of ILC3s, resulting in reduced secretion of IL‐17 and IL‐22.^[^ ^56^ ^]^

The major subpopulations of ILCs in murine lungs was ILC2s, whereas the prominent cell group in human lungs is ILC3s—which account for ∼60% of total human lung ILCs.^[^ ^57^ ^]^ ILC2s play important roles in innate immunity and disease progression, by secreting type 2 cytokines and growth factors. Additionally, ILC3‐like cells have been detected in the BALF of asthma patients.^[^ ^57^ ^]^ Lung‐resident ILCs can be maintained locally and expanded under certain physiological or pathological conditions. In addition, the hematogenous recruitment and redistribution of ILCs increases in response to severe environmental challenges during pulmonary infections.^[^ ^58^ ^]^

### 3.5 T Cells

Lung‐resident memory T cells serve as sentinels of the secondary immune response. During the activation phase of infection, DCs present antigens to activate naïve T cells in the lymph nodes, transforming them into effector T cells and upregulating surface markers such as CXCR3, CXCR6, and CCR5, which guide their entry into inflamed tissues.^[^ ^59^ ^]^ Some of these effector T cells, upon entering the lung tissue, are regulated by environmental signals—cytokines such as TGF‐β and homologous antigens, and differentiate into lung‐resident memory T cells.^[^ ^59^ ^]^ Most of these cells differentiate from effector T cells, forming a specific niche within the lungs and residing there permanently. When reinfection occurs, these pathogen‐specific resident memory T cells can control acute reinfections and contribute to long‐term immunity.^[^ ^60^ ^]^ The transcription factor Notch is an important factor in regulating the basic metabolic functions of lung‐resident memory T cells, and the loss of Notch can lead to a significant reduction in their numbers.^[^ ^61^ ^]^ The expression of IFITM3 by lung‐resident memory T cells can protect them from secondary infections and enhance their survival.^[^ ^62^ ^]^ However, lung‐resident memory T cells gradually disappear 4–5 months after infection and cannot persist like resident memory T cells in other tissues, which can exist for a long time or even a lifetime.^[^ ^59^ ^]^ Airway resident memory T cells are primarily recruited and replaced by lung interstitial resident memory T, which are continuously replenished by circulating effector memory T cells.^[^ ^63^ ^]^

The γδ T cell type represents a subset of unconventional and innate‐like T cells, showing the characteristics of both innate and adaptive immune cells.^[^ ^64^ ^]^ In addition, γδ T cells can maintain pulmonary homeostasis and participate in protective immunity against pathogens, tumor surveillance, and the regulation of innate and adaptive immune responses. The lungs represent the preferred site of perinatal γδ T cell homing. These cells are present in all areas of the lungs, except the airway mucosa.^[^ ^65^ ^]^ The γδ T cells in the lung epithelium mainly express Vδ6/Vδ1.^[^ ^66^ ^]^ After birth, the activation pattern of the Vγ gene in lung resident γδ T cells change with age. From birth until 8–10 weeks, Vγ 6 ^+^ γδ T cells represent the dominant population, while Vγ 4^+^ ones become the main population aged ∼18 years.^[^ ^65^, ^67^ ^]^ Other subsets defined by Vγ gene, such as Vγ1^+^ CD T cells, represent minority cell types in normal lungs.^[^ ^65^ ^]^ It has been noted that the distribution of Vγ4^+^ and Vγ1^+^ populations in the lung is more localized to the parenchyma.^[^ ^68^ ^]^ Studies have shown that invariant Vγ1^+^ T cells play key roles in the expansion of γδ T cells, as well as in their interactions with other γδ T cells in the lungs.^[^ ^65^, ^69^ ^]^

### 3.6 Eosinophils

Eosinophils are generally considered pro‐inflammatory cells, towing to their intracellular contents of abundant cytotoxic mediators such as eosinophil peroxidase.^[^ ^70^ ^]^ Mesnil et al. have identified a noteworthy group of lung‐resident eosinophils that expressed the surface membrane markers L‐selectin receptor CD62L and the inhibitory receptor Siglec‐F, but their expression levels differed from those of inflammatory eosinophils characterized by the Siglec‐F^hi^ CD101^hi^ CD62L^lo^ phenotype.^[^ ^71^ ^]^ Inflammatory eosinophils mainly distributed around the bronchi, whereas resident eosinophils are located in the lung parenchyma. Resident eosinophils are not present at birth, but migrate to their adult levels within 7 days following birth, which is consistent with the unique regulation of resident eosinophils.^[^ ^72^ ^]^ Experiments in eosinophil‐deficient mice have shown that resident eosinophils can promote the development of Th1‐biased immunity by inhibiting the functions of T cells or DCs, indicating that resident eosinophils have a certain degree of immunoregulatory function.^[^ ^71^ ^]^ It is particularly noteworthy that these convincing findings are mainly confined to mice—although some preliminary evidence does suggest the possible presence of resident eosinophils in human lungs as well.

### 3.7 Basophils

Basophils are short‐lived granulocytes that primarily induce Th2‐biased response during allergic reactions and IL‐4 secretion during helminth infections.^[^ ^73^ ^]^ However, their roles in tissue development and homeostasis remains unclear. Lung basophils have been shown to express various cytokines and growth factors that are essential for both immune and non‐immune cell functions.^[^ ^10^ ^]^ Owing to similarities in morphology and gene expression, mast cells and basophils are also similar in many functional aspects, primarily regarding their involvements in allergic reactions.^[^ ^74^ ^]^ Mast cells enter and complete their development in immature tissues, whereas basophils are fully differentiated in hematopoietic tissues and circulate in the blood until they are pathologically eliminated or recruited to tissues.^[^ ^75^ ^]^

Pulmonary resident basophils are located in the thinner lung tissues, particularly near to alveoli.^[^ ^10^ ^]^ In contrast to circulating basophils, resident basophils acquire unique and persistent lung signature signals and genetic patterns. The gene expression profile of pulmonary basophils differs from that of circulating basophils, characterized by the unique expression of *Il6*, *Il13*, *Cxcl2*, *Tnf*, *Osm*, and *Ccl4*. This unique genetic signature persists in adult lung‐resident basophils.^[^ ^10^ ^]^

### 3.8 B Cells

It is generally believed that memory B cells are circulating cells in the blood that can reside in inflammatory tissue for extended periods.^[^ ^76^ ^]^ A recent study found that when influenza infection occurs in the lungs, a group of tissue resident memory B cells, that stable in the lungs and have different transcriptional characteristics from those of classical memory B cells in the blood or spleen, appear in bronchus‐associated lymphoid tissues. The localization mechanism of these cells is evolutionarily conserved and may constitute an independent component of B cell immunity.^[^ ^77^ ^]^ This allows rapid humoral immunity to be mobilized when respiratory viruses invade the lungs.

## 4 Recruited Cells and Lung Microenvironment‐Determined Remodeling of New Subtypes

### 4.1 Monocyte‐Derived Macrophages

During lung infection or injury, AT2s produce chemokines and cytokines, such as CCL2 and IL‐33, which recruit monocytes to the alveolar lumen. These monocytes then differentiate into monocyte‐derived macrophages.^[^ ^78^ ^]^ During the initial phase of inflammation, these recruited cells significantly differ from lung‐resident AMs in terms of their phenotypes and metabolic characteristics. Monocyte‐derived macrophages appear to be more responsive and plastic than resident AMs.^[^ ^79^ ^]^ In response to inflammation or injury, resident AMs, epithelial cells and other innate immune cells release cytokines and chemokines that recruit classical monocytes to migrate into the lungs. These monocytes subsequently differentiate into macrophages. Monocyte‐derived macrophages have limited self‐maintenance ability, and some may undergo apoptosis during the late stages of inflammation. However, a minority of them are undergoes remodeling in the lungs to acquire the AM phenotype.^[^ ^79^, ^80^ ^]^ Due to their monocyte origin, monocyte‐derived macrophages are more readily influenced by the microenvironment and can quickly adopt an inflammatory phenotype that responds to the lung's inflammatory state, potentially further promoting inflammation.^[^ ^81^ ^]^

Studies have shown that monocyte‐derived macrophages aggravate type II inflammation.^[^ ^81^, ^82^ ^]^ However, Machiels et al. demonstrated that monocyte‐derived macrophages play an active role in type II inflammation after long‐term lung immune training.^[^ ^83^ ^]^ Recruited monocyte‐derived macrophages are more likely to acquire a fibrotic phenotype and promote pulmonary fibrosis. One study found that during pulmonary fibrosis, AMs are partially consumed and replaced by monocyte‐derived macrophages that persist in the lungs and cause pulmonary fibrosis.^[^ ^80^ ^]^ The self‐maintenance and persistence of these pathogenic monocyte‐derived macrophages are regulated by the macrophage colony stimulating factor receptor signaling pathway.^[^ ^84^ ^]^ During viral and bacterial pulmonary infections, monocyte‐derived macrophages play an important role in eliminating pathogens through phagocytosis and inflammation. However, the strong pro‐inflammatory activation of newly recruited monocyte‐derived macrophages often aggravates lung injury. Monocyte‐derived macrophages cause alveolar epithelial cell apoptosis and lung injury by releasing tumor necrosis factor‐related apoptosis‐inducing ligands and proinflammatory cytokines. The increased number of monocyte‐derived macrophages in the lungs of patients with COVID‐19 may be related to cytokine storms and tissue damages.^[^ ^85^ ^]^ In the later stage of infection, inflammation gradually subsides, and pro‐inflammatory monocyte‐derived macrophages transform to take on a repair phenotype, which in turn promotes the regression of inflammation.^[^ ^86^ ^]^ However, the mechanisms by which inflammatory monocyte‐derived macrophages gradually disappear from the alveoli (i.e., whether apoptosis or migration occurs), and exactly how pro‐inflammatory monocyte‐derived macrophages transform to acquire the repair‐promoting phenotypes, remain unclear. After recover from infection, some monocyte‐derived macrophages replenish the AM pool. These monocyte‐derived macrophages exhibit unique functional, transcriptional, and epigenetic characteristics that produce levels of increased IL‐6 and protect the lungs from subsequent attacks by Streptococcus pneumoniae.^[^ ^87^ ^]^

### 4.2 Neutrophils

The classical recruitment cascades of neutrophils into inflammatory tissues include capturing, scrolling, slow scrolling, arrest, post‐adhesion strengthening, crawling and migration.^[^ ^88^ ^]^ Neutrophils are the first immune cells to be recruited to sites of injury or inflammation. Following activation, they emerge from the vascular system and migrate to the alveolar airspace across the interstitium. Notably, neutrophil recruitment into the lung occurs in the small capillaries in a sequence of activation, sequestration from blood to interstitium and transepithelial migration. In particular, neutrophils (6–10 µm) have to change their shapes to pass through small pulmonary capillaries (2–15 µm in diameter).^[^ ^89^ ^]^

Neutrophil recruitment is considered a central factor in the development of acute lung injury (ALI) and acute respiratory distress syndrome (ARDS), because neutrophil activation and migration are landmark events in the progress of both conditions.^[^ ^90^ ^]^ In patients with ARDS, the concentration of neutrophils in the BALF is associated with the severity and prognosis of ARDS, while neutrophil depletion can inhibit the development of ALI in mice.^[^ ^91^ ^]^

The alterations in neutrophil deformability, which result from the formation of actin fibers and the rearrangement of the cytoskeleton following neutrophil activation, are deemed crucial for the recruitment of neutrophils from the marginal pool to the lungs during ALI.^[^ ^92^ ^]^ The inhibition of actin fiber formation could potentially diminish neutrophil recruitment during ALI,^[^ ^93^ ^]^ indicating that the distinctive anatomical microstructure of lung tissue plays a pivotal role in dictating neutrophil recruitment.

### 4.3 Memory T Cell

Memory T cells are a key component of the adaptive immune system, able to quickly recognize and respond to previously encountered pathogens and provide a faster and more effective immune response than an initial infection.^[^ ^94^ ^]^ In addition to the tissue‐resident T cells mentioned above, memory T cells also include central memory T cells that stimulate circulation in the blood and lymphoid organs and effector memory T cells that can migrate from the blood to non‐lymphoid tissues.^[^ ^95^ ^]^ Experimental models of respiratory viral infection showed that memory T cells were established in both lymphatic and peripheral sites, including parenchyma and airway.^[^ ^96^ ^]^ The ratio of central memory cells to effector memory cells established after infection is not static, but gradually changes to a central memory phenotype over time.^[^ ^97^ ^]^ Interestingly, in both inflammatory and homeostasis conditions, memory T cells in the airway of the lung are only effector memory phenotypes, which may explain why the number of memory T cells in the airway steadily declines during the first 6 months after infection, as does the overall effector memory cell proportion.^[^ ^98^ ^]^ Leukocyte function‐associated antigen‐1 (LFA‐1) and P‐selectin glycoprotein ligand‐1 regulate T cell recruitment to lung tissue, and LFA‐1 plays an important role in the migration of effector T cells from pulmonary vessels to non‐inflammatory lung tissue.^[^ ^99^ ^]^ Collagen‐binding integrin very late antigen −1 plays a role in the accumulation of flu‐specific CD8 T cells in the airway of the lung.^[^ ^100^ ^]^ While these molecules are important in the migration of memory T cells from the circulatory system to the lungs, almost all memory T cells upregulate these adhesion molecules in response to respiratory and non‐respiratory viral infections. In addition, the inflammatory chemokine CCL5 is highly expressed on activated T cells by binding to its receptor CCR5, guiding the migration of effector T cells from pulmonary blood vessels to surrounding tissues.^[^ ^101^ ^]^ Several molecules that affect T cell recruitment to the lungs have been identified, yet the unique phenotype of this subpopulation, particularly in terms of pulmonary airway homing, remain identified.^[^ ^99^ ^]^ Identifying the various memory cell subpopulations that contribute to this population, as well as the mechanisms governing their recruitment, could potentially assist in the development of vaccines that effectively enhance cell‐mediated immunity against respiratory viruses.

## 5 Formation of the Immune Niche Induced by Crosstalk Between Lung Immune and Tissue Cells

### 5.1 Crosstalk Between AMs and Lung Epithelial Cells

The airway epithelium serves as a robust barrier, separating the internal milieu from the external environment. The effectiveness of the pulmonary epithelial barrier is attributed to its ability to form tight apical junctions with inferior adherent junction.^[^ ^102^ ^]^ These intercellular junctions not only establish cell polarity but also act as selective permeability barriers, meticulously regulating the transit of ions and macromolecules between the apical and basolateral compartments of the epithelium.^[^ ^103^ ^]^ Lung epithelial cells play a crucial role in promoting local immune responses by generating a variety of regulatory mediators. Viral stimulation of pattern recognition receptors in lung epithelial cells induces the production of type I and type III interferons, which in turn induces the expression of hundreds of interferon‐stimulating genes. Some of these have localized direct antiviral effects, whereas others promote cellular immunity.^[^ ^104^ ^]^ Furthermore, pulmonary epithelial cells contribute to inflammation and the regulation of airway remodeling through the secretion of anti‐inflammatory factors, including IL‐10, TGF‐β, and lipoxins. However, dysregulated secretion of pro‐inflammatory cytokines by these cells can result in pulmonary pathologies such as allergic pneumonia and chronic obstructive pulmonary disease.^[^ ^105^ ^]^

**Figure 3:**
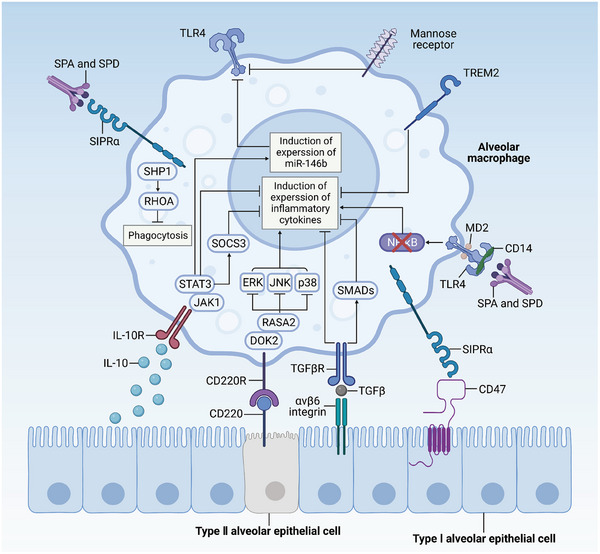
Interaction between alveolar macrophages and epithelial cells. Interleukin‐10 (IL‐10), plentiful in lung tissue, stimulates the production of inhibitory regulators like cytokine signal transduction inhibitor 3 (SOCS3) and the microRNA miR‐146b. This stimulation is mediated through the activation of the JAK1 signal transduction and STAT3 transcription pathways, which in turn suppresses inflammation. SOCS3 is instrumental in curbing the expression of pro‐inflammatory cytokines, while miR‐146b specifically hinders the expression and signaling of toll‐like receptor 4 (TLR4). Transforming growth factor‐β (TGF‐β) plays a role in modulating inflammatory responses via both dependent and independent signaling routes. The integrin αvβ6, predominantly found in bronchial epithelial cells but can also present in inflamed alveolar epithelial cells, binds to the latent TGF‐β. This binding induces a conformational change in TGF‐β, promoting its interaction with the TGF‐β receptor (TGFβR). The receptor TREM2, expressed in myeloid cells, is activated by the binding of DNAX‐activating protein 12 (DAP12). It helps to limit macrophage inflammation by interacting with one or more unidentified ligands. Mannose receptors serve to impede the recognition of TLR4 ligands, thereby restricting the phagocytosis of pathogens. The CD200 receptor (CD200R), upon binding to CD200 present on epithelial cells, triggers docking protein 2 (DOK2) and RAS GTPase activating protein RASA2. This interaction inhibits the inflammatory pathways, including extracellular signal‐regulated kinase (ERK), p38 mitogen‐activated protein kinase (MAPK), and Jun N‐terminal kinase (JNK). Pulmonary surfactant‐associated proteins A (SPA) and D (SPD), extensively found in the alveoli, prevent the interaction of TLR4 with its ligands as well as with TLRMD2 and CD14. This interaction inhibits the activation of nuclear factor‐κB (NF‐κB) and the initiation of binding of surfactant proteins to signal‐regulatory protein‐α (SIRPα). This process ultimately suppresses the recruitment of protein tyrosine phosphatase 1 (SHP1) containing the SH2 domain and the activation of RHOA—ultimately inhibiting phagocytosis.^[^ ^2^, ^111^ ^]^ Adapted with permission.^[^ ^2^ ^]^ Copyright 2014, Springer Nature. The figure was created with BioRender.com (https://biorender.com).

Pulmonary epithelial cells are pivotal in orchestrating local immune responses through their dynamic interactions with immune cells, notably AMs (as depicted in **Figure** **3**). These epithelial cells express surface molecules like CD200, CD47, and programmed death‐ligand 1 (PD‐L1), which serve as key regulators of immune cell function. Specifically, they modulate the activity of immune cells by engaging with receptors such as CD200R, SIRPα, and programmed cell death protein 1 (PD1), respectively.^[^ ^106^ ^]^ The liaison between CD200 and CD200R exerts a dampening effect on the release of inflammatory factors, thereby curbing excessive inflammation. Similarly, the interaction of the macrophage receptor SIRPα with CD47 thwarts macrophage phagocytosis, while the union of PD‐L1 with PD1 quells the production of inflammatory cytokines and tempers the effector functions of T cells within AMs.^[^ ^107^ ^]^ This intricate interplay underscores the capacity of pulmonary epithelial cells to engage in bidirectional communication with immune cells, thereby critically influencing the modulation of local immune responses.

As the frontline defenders in the lung, pulmonary epithelial cells and AMs must work in concert to neutralize invading pathogens efficiently without inflicting undue harm on the lung tissue. This coordination is achieved through direct cell‐to‐cell interactions or by the secretion of signaling molecules (as depicted in Figure 3). Under normal conditions, epithelial cells keep AMs in a quiescent state, poised for action. The interaction between AMs and epithelial cells (Figure 3) is facilitated by extracellular membrane proteins such as CD200R, PD‐1, and SIRPα on AMs, which bind to their respective ligands—CD200, PD‐L1, and CD47—on epithelial cells.^[^ ^106^, ^108^ ^]^ These protein‐protein interactions not only reinforce cellular adhesion to enhance paracrine signaling but also serve to suppress the activation of AMs. Furthermore, the proximity of these two cell types allows pulmonary endothelial cells to modulate AM behavior through the secretion of anti‐inflammatory cytokines, including IL‐10 and TGF‐β.^[^ ^105^, ^109^ ^]^ Any disruption of these regulatory interactions, whether due to epithelial damage or the detection of pathogens in the respiratory tract, triggers the activation of AMs and initiates an inflammatory cascade. While it is still a matter of debate whether epithelial cells are the primary initiators of pre‐inflammatory responses in AMs, it is clear that both cell types are indispensable for mounting an effective immune response.^[^ ^109^ ^]^

Gap junctions, which are typically intercellular contacts between epithelial cells, have an expanded role, as they also facilitate communication between AMs and alveolar epithelial cells. In mice, it has been observed that AMs express connexin 43, a protein that forms gap junctions with epithelial cells.^[^ ^18a^ ^]^ This interplay allows for the exchange of calcium (Ca^2+^) signaling between AMs and epithelial cells, reinforcing the concept of a bidirectional anti‐inflammatory dynamic within the AM‐epithelial interface. Furthermore, connexin 43 has also been identified in the interactions between macrophages and epithelial cells in humans, indicating a potential role in mediating lung cell communication across species.^[^ ^18^, ^110^ ^]^ This discovery suggests that the mechanisms underlying cellular dialogue in the lung may be conserved, highlighting the importance of gap junctions in the immune and physiological responses of the respiratory system.

### 5.2 Crosstalk Between DCs and Lung Epithelial Cells

**Figure 4:**
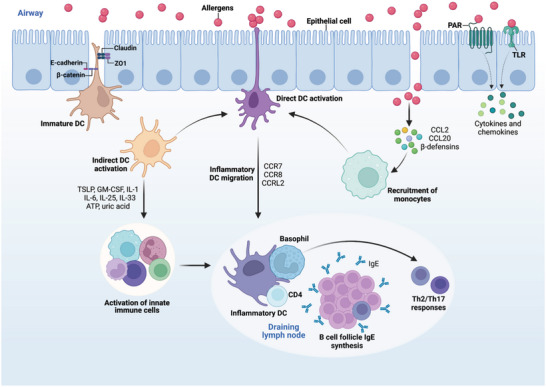
Interaction between lung dendritic cells and lung epithelial cells. Lung dendritic cells (DCs) and epithelial cells possess pattern recognition receptors (PRRs), which allergens can activate directly. Upon allergen exposure, these epithelial cells release chemokines that draw in immature conventional DCs and inflammatory monocytes, notably through the action of CCL2 and CCL20. The activation of epithelial cells also leads to the generation of cytokines (such as IL‐1, GM‐CSF, and IL‐33) and alarm signals (like ATP and uric acid), which promote the maturation of DCs. Once activated, pulmonary DCs migrate to the nearby mediastinal lymph nodes, where they play a pivotal role in initiating Th2 and Th17 responses, critical for allergic inflammation. In addition, DCs receive help from basophils to maintain the Th2 response.^[^ ^28^, ^45^, ^119^ ^]^ The figure was created with BioRender.com (https://biorender.com).

Barrier epithelial cells are the sentinels of the respiratory system, forming the initial defense against inhaled pathogens. They also express pattern recognition receptors (PRRs) such as TLRs and protease‐activated receptors (PARs), which are adept at identifying microbial motifs and allergens, respectively.^[^ ^112^ ^]^ When these receptors on epithelial cells engage with their targets, they initiate a cascade of responses that culminate in the production of chemokines. These chemokines are crucial for recruiting DCs and cytokines that foster their maturation, as illustrated in **Figure** **4**. When exposed to allergens, these epithelial cells respond by releasing chemokines, notably CCL2 and CCL20, that attract immature cDCs and inflammatory monocytes to the site of exposure. This recruitment is a critical first step in the immune response.^[^ ^113^ ^]^ Moreover, the activation of epithelial cells triggers the production of cytokines, including IL‐1, GM‐CSF, and IL‐33, along with alarm signals such as ATP and uric acid. These molecules are instrumental in the maturation process of DCs. Once activated, pulmonary DCs migrate to the nearby mediastinal lymph nodes, where they play a pivotal role in initiating Th2 and Th17 immune responses, critical for allergic inflammation.^[^ ^113^ ^]^ Research has demonstrated that the innate immune response, particularly the reaction of epithelial cells to lipopolysaccharide, is vital for amplifying the Th2 cell response in DCs.^[^ ^114^ ^]^ Th2 cells, along with eosinophils, mast cells, and basophils, play a central role in chronic respiratory inflammation. They produce mediators that not only sustain the development of DC‐driven Th2 cells but also release the signature cytokines IL‐4 and IL‐13.^[^ ^115^ ^]^ There is substantial evidence to suggest that IL‐4 receptor α, present on pulmonary DCs and lung epithelial cells, acts as a critical feedback mechanism. This mechanism allows cells producing IL‐4 and IL‐13 to further enhance Th2 cell polarization in response to allergens.^[^ ^116^ ^]^ Moreover, the interaction of IL‐4 and IL‐13 with allergens or TLR agonists stimulates lung epithelial cells to produce TSLP, GM‐CSF, and the chemokine CCL20.^[^ ^117^ ^]^ Thus, effector cells of asthma may maintain Th2 cell‐mediated inflammation directly, through the activation and recruitment of DCs, or indirectly, through the regulation of cross‐talk between epithelial cells and DCs.^[^ ^112b^ ^]^

Both In vitro and in vivo experiments have shown that allergen‐activated airway epithelial cells suggest that DCs promote Th2 sensitization.^[^ ^118^ ^]^ Respiratory epithelial cells exposed to allergens produce chemokines CCL2 and CCL20, which attract immature DCs and their precursors. Exposure experiments in TLR4^−/−^ chimeric mice have confirmed that responses to allergens changed when epithelial cells were unable to sense endotoxins in the allergens. In the lungs, both DCs and epithelial cells express a series of PRRs to sense the presence of inhaled antigens, and many of the functions of DCs are controlled by factors that are produced by epithelial cells.^[^ ^118a,b^ ^]^

### 5.3 Crosstalk Between Other Immune Cells and Lung Fibroblasts

Fibroblast function and behavior are regulated by both biochemical and physical signals. Influenza A virus (IVA) infection can cause abnormal remodeling of the lung parenchyma, depending on the severity of the infection.^[^ ^120^ ^]^ The lung extracellular matrix (ECM) represents a reservoir of many growth factors and cytokines, that are critical to cell differentiation and proliferation. During states of infection, lung fibroblasts respond to cytokines secreted by damaged epithelial cells and, in return, activated inflammatory factors remodel the ECM to create a tissue environment that promotes immune responses against infection. Boyd et al. identified two new sub‐groups of inflammatory fibroblasts.^[^ ^118a^ ^]^ Ten days after infection, when the responses of antiviral T cells reach their peak, damage‐reactive fibroblasts expressing the metalloproteinase ADAMTS4 have been found in the distal airway interstitial inflammatory area upon IVA infection and enriched in fibrotic human lungs. A transwell assay showed that the presence of ECM protease ADAMTS4 increased the migration of CD8^+^ T cells through versican‐coated membranes, indicating the increased risk of diseases in vivo.^[^ ^121^ ^]^ By contrast, in the absence of fibroblast‐derived ADAMTS4, fewer CD8^+^ T cells and higher versican levels have been observed in the lungs. These results suggest that fibroblast‐mediated modification of the ECM is involved in regulating T‐cell migration.

Some studies have found that mast cells and trypsin enhance the migratory abilities of fibroblasts, while PAR2 antagonists can weaken this ability, supporting the notion that PAR2 activation is related to the fibroblasts‐inducted migration of immune cells.^[^ ^122^ ^]^ Notably, the mast cell proteases trypsin and chymotrypsin appeared to have opposite effects regarding the release of fibroblasts in healthy states versus idiopathic fibrosis.^[^ ^123^ ^]^ Trypsin increased the release of IL‐6, vascular endothelial growth factor (VEGF), and hepatocyte growth factor from healthy fibroblasts, whereas the fibroblast response to idiopathic pulmonary fibrosis was weak. This was because chymase seemed to inhibit the release of VEGF—particularly hepatocyte growth factor.^[^ ^124^ ^]^ This evidence suggests that fibroblast‐derived trypsin promotes the migration of mast cells through the epithelial barrier.

During tissue homeostasis and damage repair in various types of immune diseases, structural stromal cells such as fibroblasts play important roles in guiding the development, survival, transport, and functions of immune cells.^[^ ^125^ ^]^ For example, a study revealed reprogramming of a variety of BM cells was mediated by COX‐2 fibroblasts in the pre‐lung metastasis niche, suggesting that targeting organ stromal cells may reshape the immune microenvironment more effectively than targeting a single BM cell population. The study found that exogenously implanted myeloid cells became dysfunctional or immunosuppressed only in the lungs, not in several other tissues that were examined. This suggests that immune cells can be uniquely reprogrammed, depending on the tissue environment they are introduced to.^[^ ^126^ ^]^ It is therefore expected that matrix‐immune cell interaction will attract increasing attention in immunological research.

## 6 New Techniques for Identification of New Sub‐Populations of Immune Cells and Relative Immune Niche

Recent studies have shown that transcriptional variation among cells can be quite high, even between genetically identical cell populations.^[^ ^127^ ^]^ Immune cells exhibit a high degree heterogeneous, which enables them to effectively combat various pathogens and diseases. This heterogeneity is often defined by the presence of specific surface markers, as identified through analysis using flow cytometry or mass‐cytometry. However, defining these markers is crucial, and their relative abundances determines the accuracy of the analysis. Therefore, increasing evidence suggests that individual single cells should be classified by transcriptome analysis and not by surface markers alone. These redefined cell types reveal the extreme heterogeneity of immune cells at the functional and genetic levels.

**Figure 5:**
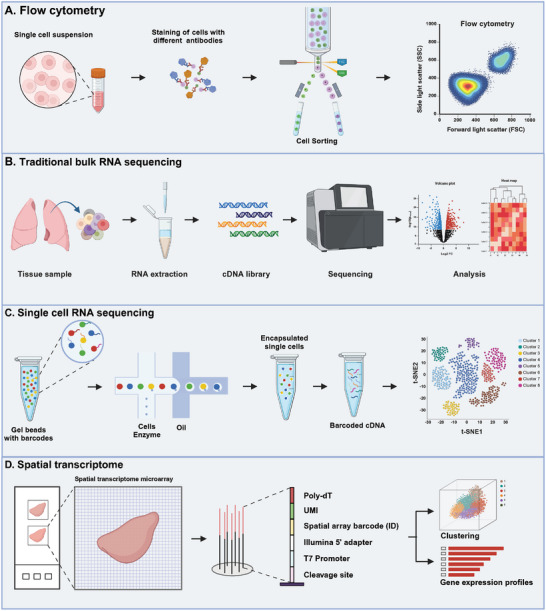
Common techniques used for identifying new lung sub‐populations include flow cytometry (A), traditional bulk RNA sequencing (B), single cell RNA sequencing (C) and spatial transcriptome (D). The figure was created with BioRender.com (https://biorender.com).

Currently, techniques such as flow cytometry, traditional bulk RNA sequencing, scRNA‐seq and spatial transcriptome (ST) analysis (**Figure** **5**), as well as combinations of two or more of these techniques, are commonly employed to identify new sub‐populations of immune cells.^[^ ^128^ ^]^ However, traditional bulk RNA sequencing involves performing transcriptome sequencing on large quantities of mixed cells, yielding average levels of gene expression, which can make it difficult to identify new cells based solely on specific gene expression.^[^ ^129^ ^]^ One limitation of traditional bulk sequencing is that it obscures important cell heterogeneity,^[^ ^129^ ^]^ resulting in cellular‐level differences being overlooked—a shortcoming that no longer aligns with the demands of contemporary scientific research.^[^ ^130^ ^]^ In contrast, ST can provide gene expression data as well as spatial location information for cells within a certain region.^[^ ^131^ ^]^ However, the accuracy of this detection approach is limited; thus, the obtained data may not support proper differentiation between single cells from larger mixtures of cells.^[^ ^131^ ^]^ Flow cytometry, which relies on fluorescent antibodies to label cells, offers advantages in the qualitative and quantitative analysis of known immune cells types but is less effective for those without specific commercial markers. Furthermore, the operation of flow cytometry is complex and requires large quantities of both cells and antibodies.^[^ ^132^ ^]^

The recent advancements in scRNA‐seq technology have significantly alleviated numerous challenges in immunological research.^[^ ^133^ ^]^ This technology offers a more robust method for identifying cell types, enabling a more nuanced profiling of cellular heterogeneity without the need for pre‐existing knowledge of genes of interest. As a consequence, scRNA‐seq has swiftly become a prominent tool, boasting the distinct advantage of transcending the constraints associated with traditional bulk RNA sequencing methods. Crucially, scRNA‐seq introduces a novel avenue for the comprehensive identification and detailed characterization of various cellular subsets.^[^ ^134^ ^]^ For example, Villani et al. utilized unbiased scRNA‐seq to uncover novel types of DCs, monocytes, and progenitor cells within human peripheral blood monocytes.^[^ ^135^ ^]^

Svetoslav et al. isolated pulmonary IMs, characterized as Lin^‐^CD45^+^MerTK^+^CD64^+^SiglecF^−^CD11b^+^, using flow cytometry and then sequenced them. The sequencing data revealed two independent monocyte‐derived resident tissue macrophage populations: Lyve1^lo^MHCII^hi^ IM and Lyve1^hi^MHCII^lo^ IM, each with unique phenotypes and gene expression profiles. Results showed that the resident tissue macrophages derived from these two monocytes were of independent lineages. The Lyve1^Lo^MHCII^hi^CX3CR1^Hi^ (Lyve1^lo^MHCII^hi^) population was mainly distributed around nerves, whereas Lyve1^hi^MHCII^lo^CX3CR1^lo^ (Lyve1^hi^MHCII^lo^) macrophages were more often located close to blood vessels across tissues. Notably, similar populations exist in multiple other tissues in addition to the lungs—including the heart, dermis, omentum, and subcutaneous adipose tissue. These two types of resident tissue macrophages preferentially occupy different niches that are conserved across different tissues types.^[^ ^32^ ^]^

Aran et al. introduced a novel computational framework, termed SingleR, designed for the annotation of scRNA‐seq data by referencing bulk transcriptomes.^[^ ^136^ ^]^ This method has been used to identify macrophages in mixed lung cell samples collected from mice with bleomycin‐induced lung injury. A novel hierarchical clustering method was also developed to identify subsets of macrophages associated with different diseases. In the research group's murine model of fibrosis, newly identified macrophage subgroups exhibited gene expression profiles that differed from those of transitional macrophage monocytes and AMs. TheSiglecF^+^CX3CR^+^ transitional profibrotic macrophages, identified in this study, are located in the fibrosis niche and play a key role in promoting fibrosis in vivo.^[^ ^136^ ^]^

Joey et al. identified two long‐lived monocyte‐derived pulmonary IM subsets, CD206^+^ IM and CD206^−^ IM, which have different phenotypes and functions. They found that CD206^+^ IMs were preferentially located around the bronchi where they produced chemokines and immunosuppressive cytokines, while CD206^−^ IMs preferentially accumulated in the alveolar interstitium and exhibited the characteristics of antigen‐presenting cells. CD64^+^CD16.2^+^ monocytes derived from intravascular Ly6C^lo^ patrolling monocytes can enter the lung tissue under normal conditions, where they become precursors of CD206^−^ IMs.^[^ ^137^ ^]^ Kaminski et al.'s study, which supported the conventional view, demonstrated that lymph nodes contain heterogeneous, long‐lived resident Treg populations. Using a photoconvertible mouse model, they tracked the circulatory kinetics of Tregs within lymph nodes over the long term. They discovered that the majority of Tregs in lymph nodes are long‐lived, memory‐like cells that persist for months and increase in number with age. These resident‐memory Tregs exhibited functional heterogeneity and shared transcriptional profiles with conventional resident memory T cells.^[^ ^138^ ^]^

Although the low capture efficiency and high loss rate of scRNA‐seq limit its sensitivity and accuracy, a more complex analytical framework is emerging to facilitate the interpretation of scRNA‐seq data.^[^ ^87^ ^]^ It is also worth mentioning that pairing analysis of single cell transcriptome data with spatial information is expected to provide new insights into transcriptional dynamics in the coming years.^[^ ^88^ ^]^ Currently, scRNA‐seq techniques can provide detailed information on cell heterogeneity, but when cells are dissociated for sequencing, their specific location in the original tissue cannot be recorded, which may have implications for understanding how cells interact in the tissue and their spatial relationship. ST technology can provide spatial level information about intercellular communication, but current spatial transcriptomic methods themselves cannot provide deep transcriptomic information about precisely located single cells in tissues.^[^ ^139^ ^]^ Therefore, the effective combination of scRNA‐seq and ST can spatially map specific cell subsets during states of development and disease, and provide new insights into the mechanism underlying the synergistic formation of tissue phenotypes by these cellular subsets.

A study using a combination of scRNA‐seq and ST found that different fibroblast profiles were observed in primary and liver metastatic tumors of colorectal cancer, providing different dimensions of liver metastasis development in colorectal cancer.^[^ ^140^ ^]^ *F3* ^+^ fibroblasts are enriched in primary tumors and lead to decreased overall survival through expression of tumor factors, while *MCAM* ^+^ fibroblasts are enriched in liver metastases and may promote CD8_CXCL13 cell generation via Notch signaling pathway.^[^ ^140^ ^]^ The integrated analysis of scRNA‐seq and ST was also used to characterize the cell atlas of the invasion trajectory of lung adenocarcinoma, revealing specific cell information and spatial structure of cancer cell and tumor microenvironment subsets, which could help in the development of individualized treatment strategies for invasive lung adenocarcinoma. The results suggested that UBE2C+ cancer cell subsets increased continuously during the invasion of lung adenocarcinoma, and significantly increased in invasive adenocarcinoma. The UBE2C+ cancer cell subsets were spatially distributed in the peripheral carcinoma area of invasive adenocarcinoma, showing a more malignant phenotype.^[^ ^141^ ^]^ This study also analyzed the subpopulations of cell types in the tumor microenvironment, and found that the number of mast cells, monocytes, and lymphatic endothelial cells involved decreased throughout the course of invasive lung adenocarcinoma, while the number of NK cells and MALT B cells increased from adenocarcinoma in situ to minimally invasive adenocarcinoma, and Treg cells and secreted B cells increased from minimally invasive adenocarcinoma to invasive adenocarcinoma.^[^ ^141^ ^]^ In addition, a joint analysis of scRNA‐seq and ST revealed spatial heterogeneity of lung adenocarcinoma from mice to humans, and identified a population of alveolar intermediary cells with elevated *KRT8* expression carrying mutations in the *KRAS* oncogene, which may represent injured or mutated cells in normal‐looking lungs and can eventually transform into lung adenocarcinoma cells.^[^ ^142^ ^]^ This study determined that alveolar intermediary cells emerge after activation of the alveolar differentiation program and can act as progenitor cells of lung adenocarcinoma with *KRAS* mutant. Alveolar intermediary cells were evident in non‐malignant areas surrounding mouse and patient samples, suggesting that the early appearance of these cells may represent a “field of injury”.^[^ ^142^, ^143^ ^]^ This study provides new implications for the development of detection and prevention strategies against early‐stage lung adenocarcinoma.

In conclusion, the combined application of scRNA‐seq and ST plays an important role in disease research by providing comprehensive information on cell composition, physiology, and cell‐cell interactions. This integrated analysis is particularly helpful in revealing specific cell information and spatial structure of cell subsets in the tumor microenvironment, providing important information for the development of individualized treatment strategies for malignant tumors. At the same time, the accuracy of disease diagnosis can be significantly improved through scRNA‐seq and ST techniques. By providing detailed molecular characteristics of cells and their spatial distribution in tissues, these techniques will contribute to a deeper understanding of disease development and cell‐cell interactions. It is hoped that the challenges of resolution, sensitivity, throughput and accessibility of scRNA‐seq and ST will be overcome in the near future to provide a richer research basis for early disease detection and precise targeted therapy.

## 7 Conclusion and Perspectives

The unique microenvironment of the lungs is regulated by intricate interactions among immune cells. The importance of these cells in defending against lung diseases and infections is increasingly recognized. When extrapulmonary immune cells are recruited to the lungs, they adapt to the local microenvironment and differentiate into cellular subsets with distinct functions. A significant knowledge gap in our understanding of lung immune cells is the characterization of their subtypes and functional heterogeneity. Further research is warranted to explore how immune cells respond to disturbances in the pulmonary microenvironment, potentially revealing new functional and heterogeneous immune cell subsets. Current knowledge of immune cell functions is primarily limited to certain types of phagocytes, such as macrophages and neutrophils. Therefore, it is critical to identify new subpopulations of immune cells and to elucidate their roles. In this review, we have clarified that progenitor cells from early lung development stages and peripheral cells recruited into the lungs can be remodeled by the pulmonary microenvironment, leading to the emergence of new cell subtypes and the establishment of specific immune niches. Accordingly, greater focus should be directed toward identifying new cell types that colonize the lungs from the early developmental stages and new cellular subsets that follow BM‐derived recruitment. We advocate that future research should prioritize elucidating the formation process and characteristics of lung immune cell niches, with a particular emphasis on understanding the spatial and temporal dynamics between lung immune cells and tissue cells. The integration of scRNA‐seq data with spatial information is anticipated to uncover new immune cell subtypes and their respective niches. Furthermore, this approach is expected to shed light on the molecular mechanisms that govern immune cell‐mediated lung homeostasis.

## Conflict of Interest

The authors declare no conflict of interest.

## References

[1] S. Saluzzo , A. D. Gorki , B. M. J. Rana , R. Martins , S. Scanlon , P. Starkl , K. Lakovits , A. Hladik , A. Korosec , O. Sharif , J. M. Warszawska , H. Jolin , I. Mesteri , A. N. J. McKenzie , S. Knapp , Cell Rep. 2017, 18, 1893. 28228256 10.1016/j.celrep.2017.01.071 PMC5329122

[2] T. Hussell , T. J. Bell , Nat. Rev. Immunol. 2014, 14, 81. 24445666 10.1038/nri3600

[3] R. Domingo‐Gonzalez , F. Zanini , X. Che , M. Liu , R. C. Jones , M. A. Swift , S. R. Quake , D. N. Cornfield , C. M. Alvira , eLife 2020, 9, 56890. 10.7554/eLife.56890 PMC7358008 32484158

[4] a) T. V. Kalymbetova , B. Selvakumar , J. A. Rodríguez‐Castillo , M. Gunjak , C. Malainou , M. R. Heindl , A. Moiseenko , C. M. Chao , I. Vadász , K. Mayer , J. Lohmeyer , S. Bellusci , E. Böttcher‐Friebertshäuser , W. Seeger , S. Herold , R. E. Morty , J. Pathol. 2018, 245, 153. 29574785 10.1002/path.5076

[5] M. Herriges , E. E. Morrisey , Development (Cambridge, England) 2014, 141, 502. 24449833 10.1242/dev.098186 PMC3899811

[6] a) R. J. Metzger , O. D. Klein , G. R. Martin , M. A. Krasnow , Nature 2008, 453, 745. 18463632 10.1038/nature07005 PMC2892995

[7] D. E. deMello , L. M. Reid , Pediatr. Pathol. Paediatr. Pathol. Soc. 2000, 3, 439. 10.1007/s100240010090 10890928

[8] a) R. L. Sanders , R. J. Hassett , A. E. Vatter , Anat. Rec. 1980, 198, 485. 6893903 10.1002/ar.1091980310

[9] J. A. Whitsett , T. Alenghat , Nat. Immunol. 2015, 16, 27. 25521682 10.1038/ni.3045 PMC4318521

[10] M. Cohen , A. Giladi , A. D. Gorki , D. G. Solodkin , M. Zada , A. Hladik , A. Miklosi , T. M. Salame , K. B. Halpern , E. David , S. Itzkovitz , T. Harkany , S. Knapp , I. Amit , Cell 2018, 175, 1031. 30318149 10.1016/j.cell.2018.09.009

[11] J. L. Barnes , M. Yoshida , P. He , K. B. Worlock , R. G. H. Lindeboom , C. Suo , J. P. Pett , A. Wilbrey‐Clark , E. Dann , L. Mamanova , L. Richardson , K. Polanski , A. Pennycuick , J. Allen‐Hyttinen , I. T. Herczeg , R. Arzili , R. E. Hynds , V. H. Teixeira , M. Haniffa , K. Lim , D. Sun , E. L. Rawlins , A. J. Oliver , P. A. Lyons , J. C. Marioni , C. Ruhrberg , Z. K. Tuong , M. R. Clatworthy , J. L. Reading , S. M. Janes , et al., Sci. Immunol. 2023, 8, adf9988. 10.1126/sciimmunol.adf9988 PMC7615868 38100545

[12] P. He , K. Lim , D. Sun , J. P. Pett , Q. Jeng , K. Polanski , Z. Dong , L. Bolt , L. Richardson , L. Mamanova , M. Dabrowska , A. Wilbrey‐Clark , E. Madissoon , Z. K. Tuong , E. Dann , C. Suo , I. Goh , M. Yoshida , M. Z. Nikolić , S. M. Janes , X. He , R. A. Barker , S. A. Teichmann , J. C. Marioni , K. B. Meyer , E. L. Rawlins , Cell 2022, 185, 4841. 36493756 10.1016/j.cell.2022.11.005 PMC7618435

[13] A. Sountoulidis , S. M. Salas , E. Braun , C. Avenel , J. Bergenstråhle , J. Theelke , M. Vicari , P. Czarnewski , A. Liontos , X. Abalo , Ž. Andrusivová , R. Mirzazadeh , M. Asp , X. Li , L. Hu , S. Sariyar , A. Martinez Casals , B. Ayoglu , A. Firsova , J. Michaëlsson , E. Lundberg , C. Wählby , E. Sundström , S. Linnarsson , J. Lundeberg , M. Nilsson , C. Samakovlis , Nat. Cell Biol. 2023, 25, 351. 36646791 10.1038/s41556-022-01064-x PMC9928586

[14] a) S. Y. Tan , M. A. Krasnow , Development (Cambridge, England) 2016, 143, 1318 26952982 10.1242/dev.129122 PMC4852511

[15] M. Guilliams , I. De Kleer , S. Henri , S. Post , L. Vanhoutte , S. De Prijck , K. Deswarte , B. Malissen , H. Hammad , B. N. Lambrecht , J. Exp. Med. 2013, 210, 1977. 24043763 10.1084/jem.20131199 PMC3782041

[16] M. Kopf , C. Schneider , S. P. Nobs , Nat. Immunol. 2015, 16, 36. 25521683 10.1038/ni.3052

[17] a) K. Asano , N. Takahashi , M. Ushiki , M. Monya , F. Aihara , E. Kuboki , S. Moriyama , M. Iida , H. Kitamura , C. H. Qiu , T. Watanabe , M. Tanaka , Nat. Commun. 2015, 6, 7802. 26193821 10.1038/ncomms8802 PMC4518321

[18] a) K. Westphalen , G. A. Gusarova , M. N. Islam , M. Subramanian , T. S. Cohen , A. S. Prince , J. Bhattacharya , Nature 2014, 506, 503. 24463523 10.1038/nature12902 PMC4117212

[19] E. M. Todd , J. Y. Zhou , T. P. Szasz , L. E. Deady , J. A. D'Angelo , M. D. Cheung , A. H. Kim , S. C. Morley , Blood 2016, 128, 2785. 27758872 10.1182/blood-2016-03-705962 PMC5159703

[20] a) C. Schneider , S. P. Nobs , M. Kurrer , H. Rehrauer , C. Thiele , M. Kopf , Nat. Immunol. 2014, 15, 1026. 25263125 10.1038/ni.3005

[21] a) T. M. Tumpey , A. García‐Sastre , J. K. Taubenberger , P. Palese , D. E. Swayne , M. J. Pantin‐Jackwood , S. Schultz‐Cherry , A. Solórzano , N. Van Rooijen , J. M. Katz , C. F. Basler , J. Virol. 2005, 79, 14933. 16282492 10.1128/JVI.79.23.14933-14944.2005 PMC1287592

[22] a) F. F. Huang , P. F. Barnes , Y. Feng , R. Donis , Z. C. Chroneos , S. Idell , T. Allen , D. R. Perez , J. A. Whitsett , K. Dunussi‐Joannopoulos , H. Shams , Am J. Respir. Crit. Care Med. 2011, 184, 259. 21474645 10.1164/rccm.201012-2036OC PMC6938174

[23] F. Ginhoux , S. Jung , Nat. Rev. Immunol. 2014, 14, 392. 24854589 10.1038/nri3671

[24] a) T. Suzuki , P. Arumugam , T. Sakagami , N. Lachmann , C. Chalk , A. Sallese , S. Abe , C. Trapnell , B. Carey , T. Moritz , P. Malik , C. Lutzko , R. E. Wood , B. C. Trapnell , Nature 2014, 514, 450. 25274301 10.1038/nature13807 PMC4236859

[25] a) S. Ma , S. Sun , J. Li , Y. Fan , J. Qu , L. Sun , S. Wang , Y. Zhang , S. Yang , Z. Liu , Z. Wu , S. Zhang , Q. Wang , A. Zheng , S. Duo , Y. Yu , J. C. I. Belmonte , P. Chan , Q. Zhou , M. Song , W. Zhang , G. H. Liu , Cell Res. 2021, 31, 415. 32913304 10.1038/s41422-020-00412-6 PMC7483052

[26] a) E. Goldstein , W. Lippert , D. Warshauer , J. Clin. Invest. 1974, 54, 519. 4853956 10.1172/JCI107788 PMC301584

[27] a) Y. Hashimoto , T. Moki , T. Takizawa , A. Shiratsuchi , Y. Nakanishi , J. Immunol. 2007, 178, 2448. 17277152 10.4049/jimmunol.178.4.2448

[28] A. Ardain , M. J. Marakalala , A. Leslie , Immunology 2020, 159, 245. 31670391 10.1111/imm.13143 PMC7011639

[29] N. Garbi , B. N. Lambrecht , Pfluegers Arch. 2017, 469, 561. 28289977 10.1007/s00424-017-1965-3

[30] C. Sabatel , C. Radermecker , L. Fievez , G. Paulissen , S. Chakarov , C. Fernandes , S. Olivier , M. Toussaint , D. Pirottin , X. Xiao , P. Quatresooz , J. C. Sirard , D. Cataldo , L. Gillet , H. Bouabe , C. J. Desmet , F. Ginhoux , T. Marichal , F. Bureau , Immunity 2017, 46, 457. 28329706 10.1016/j.immuni.2017.02.016

[31] N. V. Serbina , E. G. Pamer , Nat. Immunol. 2006, 7, 311. 16462739 10.1038/ni1309

[32] S. Chakarov , H. Y. Lim , L. Tan , S. Y. Lim , P. See , J. Lum , X. M. Zhang , S. Foo , S. Nakamizo , K. Duan , W. T. Kong , R. Gentek , A. Balachander , D. Carbajo , C. Bleriot , B. Malleret , J. K. C. Tam , S. Baig , M. Shabeer , S. E. S. Toh , A. Schlitzer , A. Larbi , T. Marichal , B. Malissen , J. Chen , M. Poidinger , K. Kabashima , M. Bajenoff , L. G. Ng , V. Angeli , et al., Science 2019, 363, aau0964. 10.1126/science.aau0964 30872492

[33] M. Fathi , A. Johansson , M. Lundborg , L. Orre , C. M. Sköld , P. Camner , Exp. Mol. Pathol. 2001, 70, 77. 11263950 10.1006/exmp.2000.2344

[34] S. L. Gibbings , S. M. Thomas , S. M. Atif , A. L. McCubbrey , A. N. Desch , T. Danhorn , S. M. Leach , D. L. Bratton , P. M. Henson , W. J. Janssen , C. V. Jakubzick , Am. J. Respir. Cell Mol. Biol. 2017, 57, 66. 28257233 10.1165/rcmb.2016-0361OC PMC5516280

[35] a) F. Ginhoux , K. Liu , J. Helft , M. Bogunovic , M. Greter , D. Hashimoto , J. Price , N. Yin , J. Bromberg , S. A. Lira , E. R. Stanley , M. Nussenzweig , M. Merad , J. Exp. Med. 2009, 206, 3115. 20008528 10.1084/jem.20091756 PMC2806447

[36] a) S. Yi , S. D. Allen , Y. G. Liu , B. Z. Ouyang , X. Li , P. Augsornworawat , E. B. Thorp , E. A. Scott , ACS Nano 2016, 10, 11290. 27935698 10.1021/acsnano.6b06451 PMC5418862

[37] Q. Lin , H. Chauvistré , I. G. Costa , E. G. Gusmao , S. Mitzka , S. Hänzelmann , B. Baying , T. Klisch , R. Moriggl , B. Hennuy , H. Smeets , K. Hoffmann , V. Benes , K. Seré , M. Zenke , Nucleic Acids Res. 2015, 43, 9680. 26476451 10.1093/nar/gkv1056 PMC4787753

[38] a) H. S. Li , C. Y. Yang , K. C. Nallaparaju , H. Zhang , Y. J. Liu , A. W. Goldrath , S. S. Watowich , Blood 2012, 120, 4363. 23033267 10.1182/blood-2012-07-441311 PMC3507145

[39] S. H. Naik , D. Metcalf , A. van Nieuwenhuijze , I. Wicks , L. Wu , M. O'Keeffe , K. Shortman , Nat. Immunol. 2006, 7, 663. 16680143 10.1038/ni1340

[40] T. Miloud , N. Fiegler , J. Suffner , G. J. Hämmerling , N. Garbi , J. Immunol. 2012, 188, 1125. 22198954 10.4049/jimmunol.1003920

[41] M. Gilliet , A. Boonstra , C. Paturel , S. Antonenko , X. L. Xu , G. Trinchieri , A. O'Garra , Y. J. Liu , J. Exp. Med. 2002, 195, 953. 11927638 10.1084/jem.20020045 PMC2193725

[42] a) B. Fancke , M. Suter , H. Hochrein , M. O'Keeffe , Blood 2008, 111, 150. 17916748 10.1182/blood-2007-05-089292

[43] a) Y. Laouar , T. Welte , X. Y. Fu , R. A. Flavell , Immunity 2003, 19, 903. 14670306 10.1016/s1074-7613(03)00332-7

[44] a) T. S. Kim , S. A. Gorski , S. Hahn , K. M. Murphy , T. J. Braciale , Immunity 2014, 40, 400. 24631155 10.1016/j.immuni.2014.02.004 PMC4017923

[45] a) F. L. Jahnsen , D. H. Strickland , J. A. Thomas , I. T. Tobagus , S. Napoli , G. R. Zosky , D. J. Turner , P. D. Sly , P. A. Stumbles , P. G. Holt , J. Immunol. 2006, 177, 5861. 17056510 10.4049/jimmunol.177.9.5861

[46] E. E. Thornton , M. R. Looney , O. Bose , D. Sen , D. Sheppard , R. Locksley , X. Huang , M. F. Krummel , J. Exp. Med. 2012, 209, 1183. 22585735 10.1084/jem.20112667 PMC3371730

[47] a) A. Schlitzer , N. McGovern , P. Teo , T. Zelante , K. Atarashi , D. Low , A. W. Ho , P. See , A. Shin , P. S. Wasan , G. Hoeffel , B. Malleret , A. Heiseke , S. Chew , L. Jardine , H. A. Purvis , C. M. Hilkens , J. Tam , M. Poidinger , E. R. Stanley , A. B. Krug , L. Renia , B. Sivasankar , L. G. Ng , M. Collin , P. Ricciardi‐Castagnoli , K. Honda , M. Haniffa , F. Ginhoux , Immunity 2013, 38, 970. 23706669 10.1016/j.immuni.2013.04.011 PMC3666057

[48] J. H. Bernink , C. P. Peters , M. Munneke , A. A. te Velde , S. L. Meijer , K. Weijer , H. S. Hreggvidsdottir , S. E. Heinsbroek , N. Legrand , C. J. Buskens , W. A. Bemelman , J. M. Mjösberg , H. Spits , Nat. Immunol. 2013, 14, 221. 23334791 10.1038/ni.2534

[49] a) N. Marquardt , E. Kekäläinen , P. Chen , E. Kvedaraite , J. N. Wilson , M. A. Ivarsson , J. Mjösberg , L. Berglin , J. Säfholm , M. L. Manson , M. Adner , M. Al‐Ameri , P. Bergman , A. C. Orre , M. Svensson , B. Dahlén , S. E. Dahlén , H. G. Ljunggren , J. Michaëlsson , J. Allergy Clin. Immunol. 2017, 139, 1321. 27670241 10.1016/j.jaci.2016.07.043

[50] G. Gasteiger , X. Fan , S. Dikiy , S. Y. Lee , A. Y. Rudensky , Science 2015, 350, 981. 26472762 10.1126/science.aac9593 PMC4720139

[51] G. E. Cooper , K. Ostridge , S. I. Khakoo , T. M. A. Wilkinson , K. J. Staples , Front. Immunol. 2018, 9, 1671. 30079068 10.3389/fimmu.2018.01671 PMC6062652

[52] Y. Hayakawa , M. J. Smyth , J. Immunol. (Baltimore, Md. : 1950) 2006, 176, 1517. 10.4049/jimmunol.176.3.1517 16424180

[53] H. Spits , D. Artis , M. Colonna , A. Diefenbach , J. P. Di Santo , G. Eberl , S. Koyasu , R. M. Locksley , A. N. McKenzie , R. E. Mebius , F. Powrie , E. Vivier , Nat. Rev. Immunol. 2013, 13, 145. 23348417 10.1038/nri3365

[54] H. Cheng , C. Jin , J. Wu , S. Zhu , Y. J. Liu , J. Chen , Protein Cell 2017, 8, 878. 28271447 10.1007/s13238-017-0379-5 PMC5712288

[55] a) S. M. Bal , J. H. Bernink , M. Nagasawa , J. Groot , M. M. Shikhagaie , K. Golebski , C. M. van Drunen , R. Lutter , R. E. Jonkers , P. Hombrink , M. Bruchard , J. Villaudy , J. M. Munneke , W. Fokkens , J. S. Erjefält , H. Spits , X. R. Ros , Nat. Immunol. 2016, 17, 636. 27111145 10.1038/ni.3444

[56] a) C. Seillet , G. T. Belz , L. A. Mielke , Cytokine 2014, 70, 1. 24972988 10.1016/j.cyto.2014.06.002

[57] K. C. De Grove , S. Provoost , F. M. Verhamme , K. R. Bracke , G. F. Joos , T. Maes , G. G. Brusselle , PLoS One 2016, 11, 0145961. 10.1371/journal.pone.0145961 PMC4699688 26727464

[58] J. Schyns , F. Bureau , T. Marichal , J. Immunol. Res. 2018, 2018, 5160794. 29854841 10.1155/2018/5160794 PMC5952507

[59] Y. Qian , Y. Zhu , Y. Li , B. Li , Front. Immunol. 2020, 11, 624411. 33603755 10.3389/fimmu.2020.624411 PMC7884312

[60] R. Channappanavar , C. Fett , J. Zhao , D. K. Meyerholz , S. Perlman , J. Virol. 2014, 88, 11034. 25056892 10.1128/JVI.01505-14 PMC4178831

[61] P. Hombrink , C. Helbig , R. A. Backer , B. Piet , A. E. Oja , R. Stark , G. Brasser , A. Jongejan , R. E. Jonkers , B. Nota , O. Basak , H. C. Clevers , P. D. Moerland , D. Amsen , R. A. van Lier , Nat. Immunol. 2016, 17, 1467. 27776108 10.1038/ni.3589

[62] L. M. Wakim , N. Gupta , J. D. Mintern , J. A. Villadangos , Nat. Immunol. 2013, 14, 238. 23354485 10.1038/ni.2525

[63] K. H. Ely , T. Cookenham , A. D. Roberts , D. L. Woodland , J. Immunol. 2006, 176, 537. 16365448 10.4049/jimmunol.176.1.537

[64] M. Bonneville , R. L. O'Brien , W. K. Born , Nat. Rev. Immunol. 2010, 10, 467. 20539306 10.1038/nri2781

[65] a) J. M. Wands , C. L. Roark , M. K. Aydintug , N. Jin , Y. S. Hahn , L. Cook , X. Yin , J. Dal Porto , M. Lahn , D. M. Hyde , E. W. Gelfand , R. J. Mason , R. L. O'Brien , W. K. Born , J. Leukocyte Biol. 2005, 78, 1086. 16204632 10.1189/jlb.0505244

[66] A. Augustin , R. T. Kubo , G. K. Sim , Nature 1989, 340, 239. 2526925 10.1038/340239a0

[67] G. K. Sim , R. Rajaserkar , M. Dessing , A. Augustin , Int. Immunol. 1994, 6, 1287. 7819138 10.1093/intimm/6.9.1287

[68] S. Li , K. Kishihara , N. Akashi , H. Hara , Y. Yoshikai , Y. Maekawa , K. Yasutomo , Biochem. Biophys. Res. Commun. 2008, 365, 246. 17981152 10.1016/j.bbrc.2007.10.163

[69] Y. Tanaka , C. T. Morita , Y. Tanaka , E. Nieves , M. B. Brenner , B. R. Bloom , Nature 1995, 375, 155. 7753173 10.1038/375155a0

[70] J. Travers , M. E. Rothenberg , Mucosal Immunol. 2015, 8, 464. 25807184 10.1038/mi.2015.2 PMC4476057

[71] C. Mesnil , S. Raulier , G. Paulissen , X. Xiao , M. A. Birrell , D. Pirottin , T. Janss , P. Starkl , E. Ramery , M. Henket , F. N. Schleich , M. Radermecker , K. Thielemans , L. Gillet , M. Thiry , M. G. Belvisi , R. Louis , C. Desmet , T. Marichal , F. Bureau , J. Clin. Invest. 2016, 126, 3279. 27548519 10.1172/JCI85664 PMC5004964

[72] M. E. Rothenberg , S. P. Hogan , Annu. Rev. Immunol. 2006, 24, 147. 16551246 10.1146/annurev.immunol.24.021605.090720

[73] a) B. Min , M. Prout , J. Hu‐Li , J. Zhu , D. Jankovic , E. S. Morgan , J. F. Urban, Jr. , A. M. Dvorak , F. D. Finkelman , G. LeGros , W. E. Paul , J. Exp. Med. 2004, 200, 507. 15314076 10.1084/jem.20040590 PMC2211939

[74] G. Varricchi , U. Raap , F. Rivellese , G. Marone , B. F. Gibbs , Immunol. Rev. 2018, 282, 8. 29431214 10.1111/imr.12627

[75] G. Marone , S. J. Galli , Y. Kitamura , Trends Immunol. 2002, 23, 425. 12200056 10.1016/s1471-4906(02)02274-3

[76] S. R. Allie , J. E. Bradley , U. Mudunuru , M. D. Schultz , B. A. Graf , F. E. Lund , T. D. Randall , Nat. Immunol. 2019, 20, 97. 30510223 10.1038/s41590-018-0260-6 PMC6392030

[77] H. X. Tan , J. A. Juno , R. Esterbauer , H. G. Kelly , K. M. Wragg , P. Konstandopoulos , S. Alcantara , C. Alvarado , R. Jones , G. Starkey , B. Z. Wang , O. Yoshino , T. Tiang , M. L. Grayson , H. Opdam , R. D'Costa , A. Vago , L. K. Mackay , C. L. Gordon , D. Masopust , J. R. Groom , S. J. Kent , A. K. Wheatley , Sci. Immunol. 2022, 7, abf5314. 10.1126/sciimmunol.abf5314 35089815

[78] F. Hou , K. Xiao , L. Tang , L. Xie , Front. Immunol. 2021, 12, 753940. 34630433 10.3389/fimmu.2021.753940 PMC8500393

[79] a) K. J. Mould , L. Barthel , M. P. Mohning , S. M. Thomas , A. L. McCubbrey , T. Danhorn , S. M. Leach , T. E. Fingerlin , B. P. O'Connor , J. A. Reisz , A. D'Alessandro , D. L. Bratton , C. V. Jakubzick , W. J. Janssen , Am. J. Respir. Cell Mol. Biol. 2017, 57, 294. 28421818 10.1165/rcmb.2017-0061OC PMC5625228

[80] J. Kulikauskaite , A. Wack , Trends Immunol. 2020, 41, 864. 32896485 10.1016/j.it.2020.08.008 PMC7472979

[81] a) X. Zhou , B. B. Moore , Eur. Respir. J. 2018, 51, 1800103; 29496789 10.1183/13993003.00103-2018 PMC6383715

[82] L. A. Borthwick , L. Barron , K. M. Hart , K. M. Vannella , R. W. Thompson , S. Oland , A. Cheever , J. Sciurba , T. R. Ramalingam , A. J. Fisher , T. A. Wynn , Mucosal Immunol. 2016, 9, 38. 25921340 10.1038/mi.2015.34 PMC4626445

[83] B. Machiels , M. Dourcy , X. Xiao , J. Javaux , C. Mesnil , C. Sabatel , D. Desmecht , F. Lallemand , P. Martinive , H. Hammad , M. Guilliams , B. Dewals , A. Vanderplasschen , B. N. Lambrecht , F. Bureau , L. Gillet , Nat. Immunol. 2017, 18, 1310. 29035391 10.1038/ni.3857

[84] N. Joshi , S. Watanabe , R. Verma , R. P. Jablonski , C. I. Chen , P. Cheresh , N. S. Markov , P. A. Reyfman , A. C. McQuattie‐Pimentel , L. Sichizya , Z. Lu , R. Piseaux‐Aillon , D. Kirchenbuechler , A. S. Flozak , C. J. Gottardi , C. M. Cuda , H. Perlman , M. Jain , D. W. Kamp , G. R. S. Budinger , A. V. Misharin , Eur. Respir. J. 2020, 55, 1900646. 31601718 10.1183/13993003.00646-2019 PMC6962769

[85] A. Bonaventura , A. Vecchié , T. S. Wang , E. Lee , P. C. Cremer , B. Carey , P. Rajendram , K. M. Hudock , L. Korbee , B. W. Van Tassell , L. Dagna , A. Abbate , Front. Immunol. 2020, 11, 1625. 32719685 10.3389/fimmu.2020.01625 PMC7348297

[86] A. C. Rothchild , G. S. Olson , J. Nemeth , L. M. Amon , D. Mai , E. S. Gold , A. H. Diercks , A. Aderem , Sci. Immunol. 2019, 4, aaw6693. 10.1126/sciimmunol.aaw6693 PMC6910245 31350281

[87] a) B. Beck‐Schimmer , R. Schwendener , T. Pasch , L. Reyes , C. Booy , R. C. Schimmer , Respir. Res. 2005, 6, 61. 15972102 10.1186/1465-9921-6-61 PMC1188075

[88] a) K. Ley , C. Laudanna , M. I. Cybulsky , S. Nourshargh , Nat. Rev. Immunol. 2007, 7, 678. 17717539 10.1038/nri2156

[89] J. Grommes , J. E. Alard , M. Drechsler , S. Wantha , M. Mörgelin , W. M. Kuebler , M. Jacobs , P. von Hundelshausen , P. Markart , M. Wygrecka , K. T. Preissner , T. M. Hackeng , R. R. Koenen , C. Weber , O. Soehnlein , Am J. Respir. Crit. Care Med. 2012, 185, 628. 22246174 10.1164/rccm.201108-1533OC PMC3326286

[90] M. A. Matthay , L. B. Ware , G. A. Zimmerman , J. Clin. Invest. 2012, 122, 2731. 22850883 10.1172/JCI60331 PMC3408735

[91] a) E. Abraham , A. Carmody , R. Shenkar , J. Arcaroli , Am. J. Physiol. Lung Cell. Mol. Physiol. 2000, 279, L1137. 11076804 10.1152/ajplung.2000.279.6.L1137

[92] a) H. Tanaka , M. Nishino , T. E. Dahms , Microvasc. Res. 2002, 63, 81. 11749075 10.1006/mvre.2001.2368

[93] G. S. Worthen , B. Schwab 3rd , E. L. Elson , G. P. Downey , Science 1989, 245, 183. 2749255 10.1126/science.2749255

[94] J. E. Kohlmeier , D. L. Woodland , Curr. Opin. Immunol. 2006, 18, 357. 16616475 10.1016/j.coi.2006.03.012

[95] a) K. K. McKinstry , T. M. Strutt , S. L. Swain , Immunology 2010, 130, 1. 20331470 10.1111/j.1365-2567.2010.03259.x PMC2855787

[96] a) D. R. Marshall , S. J. Turner , G. T. Belz , S. Wingo , S. Andreansky , M. Y. Sangster , J. M. Riberdy , T. Liu , M. Tan , P. C. Doherty , Proc. Natl. Acad. Sci. USA 2001, 98, 6313. 11344265 10.1073/pnas.101132698 PMC33465

[97] a) E. J. Wherry , V. Teichgräber , T. C. Becker , D. Masopust , S. M. Kaech , R. Antia , U. H. von Andrian , R. Ahmed , Nat. Immunol. 2003, 4, 225. 12563257 10.1038/ni889

[98] R. J. Hogan , L. S. Cauley , K. H. Ely , T. Cookenham , A. D. Roberts , J. W. Brennan , S. Monard , D. L. Woodland , J. Immunol. 2002, 169, 4976. 12391211 10.4049/jimmunol.169.9.4976

[99] a) J. C. Lehmann , D. Jablonski‐Westrich , U. Haubold , J. C. Gutierrez‐Ramos , T. Springer , A. Hamann , J. Immunol. 2003, 171, 2588. 12928410 10.4049/jimmunol.171.5.2588

[100] S. J. Ray , S. N. Franki , R. H. Pierce , S. Dimitrova , V. Koteliansky , A. G. Sprague , P. C. Doherty , A. R. de Fougerolles , D. J. Topham , Immunity 2004, 20, 167. 14975239 10.1016/s1074-7613(04)00021-4

[101] E. Galkina , J. Thatte , V. Dabak , M. B. Williams , K. Ley , T. J. Braciale , J. Clin. Invest. 2005, 115, 3473. 16308575 10.1172/JCI24482 PMC1288831

[102] S. N. Georas , F. Rezaee , J. Allergy Clin. Immunol. 2014, 134, 509. 25085341 10.1016/j.jaci.2014.05.049 PMC4170838

[103] J. Yang , W. L. Zuo , T. Fukui , I. Chao , K. Gomi , B. Lee , M. R. Staudt , R. J. Kaner , Y. Strulovici‐Barel , J. Salit , R. G. Crystal , R. Shaykhiev , Am J. Respir. Crit. Care Med. 2017, 196, 340. 28345955 10.1164/rccm.201608-1672OC PMC5549864

[104] M. C. Altman , S. R. Reeves , A. R. Parker , E. Whalen , K. M. Misura , K. A. Barrow , R. G. James , T. S. Hallstrand , S. F. Ziegler , J. S. Debley , J. Allergy Clin. Immunol. 2018, 142, 451. 29106997 10.1016/j.jaci.2017.10.004 PMC5951761

[105] a) S. Matikainen , J. Sirén , J. Tissari , V. Veckman , J. Pirhonen , M. Severa , Q. Sun , R. Lin , S. Meri , G. Uzé , J. Hiscott , I. Julkunen , J. Virol. 2006, 80, 3515. 16537619 10.1128/JVI.80.7.3515-3522.2006 PMC1440408

[106] a) Y. F. Jiang‐Shieh , H. F. Chien , C. Y. Chang , T. S. Wei , M. M. Chiu , H. M. Chen , C. H. Wu , J. Anat. 2010, 216, 407. 20070425 10.1111/j.1469-7580.2009.01190.x PMC2829398

[107] a) H. Okazawa , S. Motegi , N. Ohyama , H. Ohnishi , T. Tomizawa , Y. Kaneko , P. A. Oldenborg , O. Ishikawa , T. Matozaki , J. Immunol. 2005, 174, 2004. 15699129 10.4049/jimmunol.174.4.2004

[108] M. Oumouna , M. Weitnauer , V. Mijošek , L. M. Schmidt , T. Eigenbrod , A. H. Dalpke , Immunobiology 2015, 220, 1240. 26153873 10.1016/j.imbio.2015.06.017

[109] S. Matsukura , F. Kokubu , M. Kurokawa , M. Kawaguchi , K. Ieki , H. Kuga , M. Odaka , S. Suzuki , S. Watanabe , H. Takeuchi , T. Kasama , M. Adachi , Clin. Exp. Allergy 2006, 36, 1049. 16911361 10.1111/j.1365-2222.2006.02530.x

[110] A. Beckmann , A. Grissmer , C. Meier , T. Tschernig , Ann. Anat. 2020, 227, 151417. 31563569 10.1016/j.aanat.2019.151417

[111] E. Y. Bissonnette , J. F. Lauzon‐Joset , J. S. Debley , S. F. Ziegler , Front. Immunol. 2020, 11, 583042. 33178214 10.3389/fimmu.2020.583042 PMC7593577

[112] a) H. F. Kauffman , Clin. Rev. Allergy Immunol. 2006, 30, 129. 16645224 10.1385/criai:30:2:129

[113] a) A. Kiss , M. Montes , S. Susarla , E. A. Jaensson , S. M. Drouin , R. A. Wetsel , Z. Yao , R. Martin , N. Hamzeh , R. Adelagun , S. Amar , F. Kheradmand , D. B. Corry , J. Allergy Clin. Immunol. 2007, 120, 334. 17544098 10.1016/j.jaci.2007.04.025

[114] M. A. Nolte , S. Leibundgut‐Landmann , O. Joffre , C. Reis e Sousa , J. Exp. Med. 2007, 204, 1487. 17548522 10.1084/jem.20070325 PMC2118612

[115] D. C. Webb , Y. Cai , K. I. Matthaei , P. S. Foster , J. Immunol. 2007, 178, 219. 17182558 10.4049/jimmunol.178.1.219

[116] N. A. Gandhi , B. L. Bennett , N. M. Graham , G. Pirozzi , N. Stahl , G. D. Yancopoulos , Nat. Rev. Drug Discovery 2016, 15, 35. 26471366 10.1038/nrd4624

[117] B. Causton , R. A. Ramadas , J. L. Cho , K. Jones , A. Pardo‐Saganta , J. Rajagopal , R. J. Xavier , B. D. Medoff , J. Immunol. 2015, 195, 683. 26041536 10.4049/jimmunol.1402983 PMC4489191

[118] a) F. Kheradmand , A. Kiss , J. Xu , S. H. Lee , P. E. Kolattukudy , D. B. Corry , J. Immunol. 2002, 169, 5904. 12421974 10.4049/jimmunol.169.10.5904

[119] a) M. Willart , H. Hammad , Curr. Opin. Immunol. 2011, 23, 772. 22074731 10.1016/j.coi.2011.09.008

[120] B. W. Seymour , L. J. Gershwin , R. L. Coffman , J. Exp. Med. 1998, 187, 721. 9480982 10.1084/jem.187.5.721 PMC2212168

[121] I. Boldogh , A. Bacsi , B. K. Choudhury , N. Dharajiya , R. Alam , T. K. Hazra , S. Mitra , R. M. Goldblum , S. Sur , J. Clin. Invest. 2005, 115, 2169. 16075057 10.1172/JCI24422 PMC1180538

[122] M. Bagher , A. K. Larsson‐Callerfelt , O. Rosmark , O. Hallgren , L. Bjermer , G. Westergren‐Thorsson , Cell Commun. Signaling 2018, 16, 59. 10.1186/s12964-018-0269-3 PMC6139170 30219079

[123] M. Wygrecka , B. K. Dahal , D. Kosanovic , F. Petersen , B. Taborski , S. von Gerlach , M. Didiasova , D. Zakrzewicz , K. T. Preissner , R. T. Schermuly , P. Markart , Am. J. Pathol. 2013, 182, 2094. 23562441 10.1016/j.ajpath.2013.02.013

[124] a) G. E. Shochet , E. Brook , B. Bardenstein‐Wald , D. Shitrit , Respir. Res. 2020, 21, 56. 32070329 10.1186/s12931-020-1319-0 PMC7029598

[125] a) S. Davidson , M. Coles , T. Thomas , G. Kollias , B. Ludewig , S. Turley , M. Brenner , C. D. Buckley , Nat. Rev. Immunol. 2021, 21, 704. 33911232 10.1038/s41577-021-00540-z

[126] Z. Gong , Q. Li , J. Shi , J. Wei , P. Li , C. H. Chang , L. D. Shultz , G. Ren , Immunity 2022, 55, 1483. 35908547 10.1016/j.immuni.2022.07.001 PMC9830653

[127] a) T. Hashimshony , F. Wagner , N. Sher , I. Yanai , Cell Rep. 2012, 2, 666. 22939981 10.1016/j.celrep.2012.08.003

[128] a) D. D. Liu , J. Q. He , R. Sinha , A. E. Eastman , A. M. Toland , M. Morri , N. F. Neff , H. Vogel , N. Uchida , I. L. Weissman , Cell 2023, 186, 1179. 36931245 10.1016/j.cell.2023.02.017 PMC10409303

[129] Q. F. Wills , K. J. Livak , A. J. Tipping , T. Enver , A. J. Goldson , D. W. Sexton , C. Holmes , Nat. Biotechnol. 2013, 31, 748. 23873083 10.1038/nbt.2642

[130] C. Trapnell , D. Cacchiarelli , J. Grimsby , P. Pokharel , S. Li , M. Morse , N. J. Lennon , K. J. Livak , T. S. Mikkelsen , J. L. Rinn , Nat. Biotechnol. 2014, 32, 381. 24658644 10.1038/nbt.2859 PMC4122333

[131] X. Li , C. Y. Wang , Int. J. Oral Sci. 2021, 13, 36. 34782601 10.1038/s41368-021-00146-0 PMC8593179

[132] A. Adan , G. Alizada , Y. Kiraz , Y. Baran , A. Nalbant , Crit. Rev. Biotechnol. 2017, 37, 163. 26767547 10.3109/07388551.2015.1128876

[133] X. Wang , Y. He , Q. Zhang , X. Ren , Z. Zhang , Genomics, Proteomics, Bioinf. 2021, 19, 253. 10.1016/j.gpb.2020.02.005 PMC8602399 33662621

[134] a) S. Liu , C. Trapnell , F1000Res. 2016, 5, 182; 10.12688/f1000research.7223.1 PMC4758375 26949524

[135] A. C. Villani , R. Satija , G. Reynolds , S. Sarkizova , K. Shekhar , J. Fletcher , M. Griesbeck , A. Butler , S. Zheng , S. Lazo , L. Jardine , D. Dixon , E. Stephenson , E. Nilsson , I. Grundberg , D. McDonald , A. Filby , W. Li , P. L. De Jager , O. Rozenblatt‐Rosen , A. A. Lane , M. Haniffa , A. Regev , N. Hacohen , Science 2017, 356, aah4573. 10.1126/science.aah4573 PMC5775029 28428369

[136] D. Aran , A. P. Looney , L. Liu , E. Wu , V. Fong , A. Hsu , S. Chak , R. P. Naikawadi , P. J. Wolters , A. R. Abate , A. J. Butte , M. Bhattacharya , Nat. Immunol. 2019, 20, 163. 30643263 10.1038/s41590-018-0276-y PMC6340744

[137] J. Schyns , Q. Bai , C. Ruscitti , C. Radermecker , S. De Schepper , S. Chakarov , F. Farnir , D. Pirottin , F. Ginhoux , G. Boeckxstaens , F. Bureau , T. Marichal , Nat. Commun. 2019, 10, 3964. 31481690 10.1038/s41467-019-11843-0 PMC6722135

[138] A. Kaminski , F. T. Hager , L. Kopplin , F. Ticconi , A. Leufgen , E. Vendelova , L. Rüttger , G. Gasteiger , V. Cerovic , W. Kastenmüller , O. Pabst , M. Ugur , Sci. Immunol. 2023, 8, adj5789. 10.1126/sciimmunol.adj5789 37874251

[139] S. K. Longo , M. G. Guo , A. L. Ji , P. A. Khavari , Nat. Rev. Genet. 2021, 22, 627. 34145435 10.1038/s41576-021-00370-8 PMC9888017

[140] F. Wang , J. Long , L. Li , Z. X. Wu , T. T. Da , X. Q. Wang , C. Huang , Y. H. Jiang , X. Q. Yao , H. Q. Ma , Z. X. Lian , Z. B. Zhao , J. Cao , Sci. Adv. 2023, 9, adf5464. 10.1126/sciadv.adf5464 PMC10275599 37327339

[141] J. Zhu , Y. Fan , Y. Xiong , W. Wang , J. Chen , Y. Xia , J. Lei , L. Gong , S. Sun , T. Jiang , Exp. Mol. Med. 2022, 54, 2060. 36434043 10.1038/s12276-022-00896-9 PMC9722784

[142] G. Han , A. Sinjab , Z. Rahal , A. M. Lynch , W. Treekitkarnmongkol , Y. Liu , A. G. Serrano , J. Feng , K. Liang , K. Khan , W. Lu , S. D. Hernandez , Y. Liu , X. Cao , E. Dai , G. Pei , J. Hu , C. Abaya , L. I. Gomez‐Bolanos , F. Peng , M. Chen , E. R. Parra , T. Cascone , B. Sepesi , S. J. Moghaddam , P. Scheet , M. V. Negrao , J. V. Heymach , M. Li , S. M. Dubinett , et al., Nature 2024, 627, 656. 38418883 10.1038/s41586-024-07113-9 PMC10954546

[143] K. Steiling , J. Ryan , J. S. Brody , A. Spira , Cancer Prevent. Res. 2008, 1, 396. 10.1158/1940-6207.CAPR-08-0174 PMC2705781 19138985

